# Gasdermin E deficiency limits inflammation and lung damage during influenza virus infection

**DOI:** 10.1038/s41419-025-07748-0

**Published:** 2025-06-06

**Authors:** Sarah Rosli, Rebecca L. Ambrose, Christopher M. Harpur, Maggie Lam, Christopher Hodges, Kristian T. Barry, Alison C. West, Ashley Mansell, Kate E. Lawlor, Michelle D. Tate

**Affiliations:** 1 Centre for Innate Immunity and Infectious Diseases, Hudson Institute of Medical Research, Clayton, VIC Australia; 2 Department of Molecular and Translational Sciences, Monash University, Clayton, VIC Australia; 3 Department of Microbiology, Anatomy, Physiology and Pharmacology, La Trobe University, Bundoora, VIC Australia

**Keywords:** Influenza virus, Acute inflammation

## Abstract

Severe influenza A virus (IAV) infections are associated with hyperinflammation and significant lung damage. Gasdermin E (GSDME) mediates pyroptosis, a lytic and inflammatory type of cell death. Cleavage of GSDME by caspase-3 releases the active N-terminal domain, which subsequently forms transmembrane pores, leading to cell lysis and death. In this study, we investigated a role for GSDME in severe influenza. Infection of human bronchial epithelial cells revealed that IAV induces GSDME cleavage and activation, with the magnitude and kinetics of GSDME activation differing between IAV strains. Caspase-3-mediated GSDME activation preceded and overwhelmed gasdermin D (GSDMD) activation. siRNA silencing in vitro confirmed both gasdermins are active in human bronchial epithelial cells and cooperate to drive IAV responses. IAV infection of mice promoted GSDME cleavage in E-cadherin^+^ epithelial cells in vivo at day 3. Mice deficient in GSDME (*Gsdme*^−/−^) showed improved survival and greater influenza disease resistance compared to their wildtype littermate controls. *Gsdme*^−/−^ mice exhibited reduced neutrophil infiltration and levels of cytokines IL-6 and IL-1β in the airways and IL-6, TNF, and IFNγ in the serum. This was accompanied by reduced viral loads, lung pathology, and epithelial cell death. Together, these findings demonstrate a pivotal role for GSDME in severe influenza pathogenesis.

## Introduction

Influenza A viruses (IAV) remain a significant public health concern, causing both recurrent epidemics and sporadic pandemics [1]. While healthy individuals do recover from IAV infection, young children, the elderly, and otherwise immunocompromised individuals are at a higher risk of developing severe and life-threatening pulmonary disease [2]. Dysregulated host responses, including excessive production of pro-inflammatory cytokines and immune cell infiltration, are associated with adverse lung damage demonstrated in severe and fatal IAV infections [3]. Given that current antiviral drugs have been shown to have limited efficacy [4] and the rise in antiviral drug resistance [5], a novel host-directed therapeutic that limits inflammation and lung damage could be a viable therapeutic option to limit influenza morbidity and mortality.

Severe influenza virus infections have been associated with significant cell death within the lung [6]. Apoptosis is the most widely studied form of cell death that is generally considered to be immunologically silent. Apoptosis involves a series of caspase-mediated cleavage events that promote cell shrinkage, cell membrane blebbing, and the formation of apoptotic bodies, which are subsequently phagocytosed [7]. In contrast, pyroptosis is a highly inflammatory type of cell death characterized by the formation of transmembrane pores, cytosol swelling, and cell membrane lysis [8–10]. Critically, cell lysis allows the release of cellular contents, including pro-inflammatory cytokines, pathogen-associated molecular patterns (PAMPs), and danger-associated molecular patterns (DAMPs), that can amplify inflammation [11]. Gasdermins are a family of functionally diverse pore-forming proteins that are essential for pyroptosis. Gasdermin D (GSDMD) and gasdermin E (GSDME; also known as DFNA5) are two major effectors of pyroptosis downstream of caspase activation [8, 10, 12]. They are expressed as an inactive precursor, consisting of an inhibitory C-terminal domain and an effector N-terminal (NT) domain, with a linker region in between [13]. Proteolytic cleavage of the linker region of GSDME by apoptotic caspase-3 or GSDMD by inflammatory caspase-1 and -11 (caspase-4/5 in humans) releases active NT domains. The monomeric NT subunit localizes to the plasma cell membrane and oligomerizes to create transmembrane pores. Gasdermin pores facilitate the release of pro-inflammatory molecules, including inflammasome-dependent cytokines that lack a secretory signal peptide, such as interleukin (IL)-1β, and allow the passive influx of ions and fluid, leading to cell swelling and lysis [14, 15].

We have recently shown GSDMD plays a detrimental role during severe IAV infection [16]. Specifically, IAV infection induced cleavage of GSDMD in epithelial cells in vivo, and GSDMD-deficient mice displayed improved disease resistance, which correlated with reduced pulmonary inflammation and lung damage. However, the role of GSDME during severe IAV infection is less well described, with one recent report showing that GSDME-deficient mice have improved survival outcomes following infection with the highly pathogenic avian H7N9 IAV subtype and that in vitro infection of primary human alveolar epithelial cells and monocytes with the same virus strain induced GSDME cleavage [17]. Consequently, further work is required to establish the significance of GSDME activity in influenza pathogenesis.

In this study, we investigated a potential role for GSDME in promoting immunopathology during severe influenza infection. IAV infection of human bronchial epithelial cells induced GSDME cleavage, with the magnitude and kinetics of GSDME activation differing between IAV strains. GSDME activation occurred before GSDMD activation, with greater intensity, partly due to caspase-3-mediated inactivation events. Importantly, gene knockdown using siRNA confirmed that GSDMD and GSDME are both active in bronchial epithelial cells following IAV infection. However, significantly, suppression of GSDME but not GSDMD limited infectious virus release. Importantly, we showed that H3N2 IAV infection of mice promoted GSDME cleavage in E-cadherin^+^ epithelial cells in the lung. Mice deficient in GSDME (*Gsdme*^−/−^) displayed less severe clinical presentation and significantly improved survival outcomes, which correlated with reduced neutrophil infiltration. This was accompanied by reduced local airway and systemic levels of pro-inflammatory cytokines. In addition, GSDME deficiency reduced viral loads and lung pathology, including peribronchial inflammation, epithelial damage, and cell death.

## Materials and Methods

### Influenza virus

The IAV H3N2 strain HKx31, a high-yielding reassortment of A/Puerto Rico/8/34 A/PR/8 (PR8; H1N1) with the surfaces of A/Hong Kong/1/68 (H3N2), was used in both in vivo and in vitro models of infection. Human influenza strains A/Brazil/11/78 (BR; H1N1), A/Solomon Island/3/2006 (SI; H1N1), A/Tasmania/2004/2009 (Tas; pandemic H1N1), and A/Perth/16/2009 (Perth; H3N2) were used in in vitro experiments. IAVs were propagated in 10-day old embryonated chicken eggs, and viral stocks were stored at -80 °C. Viral titers were quantified on Madin-Darby Canine Kidney (MDCK) cells using a standard plaque assay.

### In vitro influenza virus infection

Normal immortalized human bronchial epithelial cells (HBEC3-KT; ATCC, Virginia, USA) were cultured in complete bronchial epithelial growth medium (BEGM; Lonza, Basel, Switzerland) and bovine collagen-coated flasks (Gibco, Thermo Fisher Scientific, Waltham, USA). HBEC3-KT cells in hydrocortisone-free BEGM medium were plated at 1 × 10^5^ cells/well into collagen-coated 12-well plates. Cells were incubated overnight and then infected with various strains of IAV at a multiplicity of infection (MOI) of 3 for 1 h. Supernatants were then discarded and replaced with complete BEGM. At the indicated time points, cell supernatants were harvested, and cell lysates were collected using RIPA buffer (50 mM Tris-HCl pH 8, 150 mM NaCl, 1 mM ethylenediaminetetraacetic acid (EDTA) pH 8, 1% v/v Igepal, 0.5% w/v sodium deoxycholate, 0.1% v/v SDS, 10 mM sodium fluoride, 1 mM sodium orthovanadate, 1 mM phenylmethylsulfonyl fluoride, and cOmplete Protease Inhibitor Cocktail (Roche, Basel, Switzerland)) and stored at −80 °C. Cell supernatants were assayed for levels of lactate dehydrogenase (LDH) using a CytoTox 96 Non-radioactive Cytotoxicity Assay (Promega, Madison, USA), according to the manufacturer’s instructions. Levels of IL-1β and IL-18 in cell supernatants were determined by ELISA (R&D Systems, Minneapolis, USA), according to the manufacturer’s instructions.

### Examination of protein expression by immunoblot

Supernatants from HBEC3-KT were concentrated using StrataClean Resin (Agilent, Santa Clara, USA) in loading buffer (250 mM Tris pH 6.8, 12% v/v SDS, 50% glycerol, 0.025% bromophenol blue) containing 5 mM dithiothreitol. HBEC3-KT cell lysates were sonicated and centrifuged. Protein levels were quantified using the Pierce^TM^ BCA Protein Assay Kit (Thermo Fisher Scientific) according to the manufacturer’s instructions. Proteins were separated on a 4-12% NuPAGE^TM^ Bis-Tris Protein Gel in 1X NuPAGE^TM^ MES running buffer (Thermo Fisher Scientific) and transferred onto Immobilon®-FL PVDF membrane (Merck Millipore, Massachusetts, USA). Membranes were blocked with 5% bovine serum albumin (BSA) in either TBS 0.1% Tween-20 (TBST), 5% skim milk in TBST, or Intercept (PBS) blocking buffer (LI-COR Biotech, Nebraska, USA) for 30 min and incubated overnight at 4 °C with primary antibodies: GSDME (Abcam, Cambridge, UK; EPR19859), GSDMD (Cell Signalling Technology; 69469), caspase-1 (Cell Signalling Technology; 3866 or 83383), caspase-3 (Cell Signalling Technology; 9662 or 9664), GAPDH (Cell Signalling Technology; 97166), and α-tubulin (Abcam; YL1/2). The following day, membranes were washed twice in TBST before being incubated with horseradish peroxidase (HRP), Alexa 680 (Thermo Fisher), or Dylight 800 (Rockland Immunochemicals, Pennsylvania, USA)-conjugated secondary antibodies for 2 h at room temperature. Membranes were washed with TBST before being visualized on the Bio-Rad ChemiDoc MP Imaging System (Bio-Rad, Hercules, USA) or Odyssey® LI-COR Imaging System (LI-COR Biotech) via chemiluminescence or fluorescence. All original blots are included in the Supplementary Information.

### siRNA silencing of GSDMD and GSDME in vitro

In the indicated experiments, siRNA knockdown of GSDMD and GSDME in HBEC3-KT cells was performed individually in triplicate wells. Briefly, siRNA specific to *GSDMD* (two different target sequences combined: #1, sense 5′-AGCUGGUUAUUGACUCUGAtt-3′, antisense 5′-CUGAGUCAAUAACCAGCUgg-3′; #2, sense 5′-GGAACUCGCUAUCCCUGUUtt-3′, antisense 5′-AACAGGGAUAGCGAGUUCCgg-3′; Silence Select, Ambion, Life Technologies), *GSDME* (two different target sequences combined: #1, sense 5′-GAGAGAACAAUAAAUCUGAtt-3′, antisense 5′-UCAGAUUUAUUGUUCUCUCgg-3′; #2, sense 5′-GAAAAGAUACAGAAAAGGUUUtt-3′, antisense 5′-AAACCUUUCUGUAUCUUUCag-3′; Ambion, Life Technologies) or non-targeting control siRNA (Silence Select Negative Control #1, Ambion) was transfected using Lipofectamine™ RNAiMAX Transfection Reagent (Thermo Fisher; 7.5 pM siRNA complexed with 0.75 μL RNAiMax per well). At 48 h following siRNA transfection, triplicate wells were infected with Brazil/78 or HKx31 IAV (MOI 3), as described above. At 24 h post-IAV infection, cell lysates were collected using RIPA buffer and pooled from triplicate wells. Suppression of GSDMD and/or GSDME expression was confirmed by immunoblot, as described above. Cell supernatants from each triplicate well were assayed for levels of lactate dehydrogenase (LDH) and IL-1β, as described above. Levels of infectious virus in cell supernatants from each triplicate well were determined using a standard plaque assay on MDCK cells. Data was normalized to NT control, where indicated, to control for inherent experimental variation.

### Influenza virus infection of mice

6-12-week-old male and female *Gsdme*^−/−^ mice and wildtype littermates on the C57BL/6N background were maintained in the Specific Pathogen Free Physical Containment Level 2 (PC2) Animal Research Facility at the Monash Medical Centre (Clayton, Victoria, Australia). *Gsdme*^−/−^ mice were kindly supplied by VM Dixit (Genentech, South San Francisco, USA). All procedures were approved by the Hudson Animal Ethics, and experimental procedures were carried out in accordance with approved guidelines.

For in vivo infection studies, cages of mice were randomly allocated to groups. Mice were lightly anesthetized with isoflurane and intranasally inoculated with 10^4^ plaque-forming units (PFU) of HKx31 IAV in 50 µl of PBS to induce severe influenza disease, as previously described [16, 18, 19]. Mice were weighed daily and assessed for clinical signs of disease on a scale of 0–3 (0 = no visible signs; 1 = slight ruffling of fur; 2 = ruffled fur, reduced mobility; and 3 = ruffled fur, reduced mobility, and rapid breathing). Animals that lost 20% of their original body weight and/or displayed severe clinical signs of disease (clinical score of 3) were immediately euthanized. In the survival cohort, mice were monitored for up to 10 days.

In another cohort, at the indicated time points, mice were sacrificed via intraperitoneal injection of sodium pentobarbital. Blood was immediately harvested via cardiac puncture, and serum was collected using Serum Gel microtubes (Sarstedt, Nümbrecht, Germany). Bronchoalveolar lavage (BAL) was obtained by flushing the lungs three times with 1 ml of cold PBS and was placed on ice. BAL fluid was collected following centrifugation. BAL fluid and serum were stored at −80 °C until further analysis was performed. Lung tissues were excised following BAL collection and snap-frozen in liquid nitrogen before titration on MDCK cells to measure levels of infectious virus using a standard plaque assay. A final cohort of mice was sacrificed via intraperitoneal injection of sodium pentobarbital, and lung tissues were immediately inflated and fixed with 10% neutral buffered formalin (NBF).

### Cytokine and chemokine analysis of mouse BAL fluids and serum

Levels of IL-6, CCL2, IFNγ, IL-10, IL-12p70, and TNF proteins were determined in BAL and serum by cytokine bead array (CBA) using the mouse inflammation kit (BD Biosciences, San Jose, USA). Mouse IL-1β, IL-18, IL-1α, CXCL1, and CXCL2 were quantified by ELISA (R&D Systems). Levels of IFNβ and IFNα in BAL were measured by ELISA, as previously described [20].

### Flow cytometry analysis of mouse BAL cells

Cells in the BAL were separated by centrifugation at 1600 rpm for 5 min at 4 °C. BAL cells were treated with a red blood cell lysis buffer (Sigma Aldrich, St. Louis, USA) at room temperature. After 5 min, FACS buffer (PBS containing 2% v/v fetal calf serum (FCS, Thermo Fisher Scientific) and 2 mM EDTA (Promega)) was added to quench the reaction. BAL cells were stained with fluorescently labeled antibodies in the FACS buffer, including Fc receptor-blocking monoclonal antibodies against CD16/CD32 (clone 93, Thermo Fisher Scientific) to prevent non-specific antibody binding at 4 °C for 20 min. BAL cells were also resuspended with a standard amount of blank calibration particles (ProSciTech, Kirwan, Australia) to determine cell count. The monoclonal antibodies used to stain the various infiltrated immune BAL cells are Siglec-F (clone E50-2440, BD Biosciences), NK1.1 (clone PK136, BioLegend, San Diego, USA), CD3ε (clone 145-2C11), CD11c (clone HL3, BD Biosciences), CD64 (clone X54-5/7.1 BioLegend), Ly6C (clone AL-21, BD Biosciences), Ly6G (clone 1A8, BD Biosciences), I-A^b^ (clone AF6-120.1, BD Biosciences), and the Zombie Aqua or Zombie NIR viability dye (BioLegend). Total live cells (Zombie Aqua^-^ or NIR viability dye^-^), neutrophils (Ly6G^+^ Ly6C^int^), natural killer (NK) cells (NK1.1^+^ CD3^-^), T cells (NK1.1^-^ CD3^+^), inflammatory monocytes/macrophages (IM; Ly6G^-^ Ly6C^hi^), alveolar macrophages (AM; Ly6C^int^ CD11c^+^ Siglec-F^+^), and dendritic cells (DC; CD11c^+^ I-A^b+^) were quantified by flow cytometry using an Aurora flow cytometer (Cytek Biosciences, Fremont, USA) and analyzed using FlowJo^TM^ 10 analysis software (BD Biosciences). Cells were enumerated using a standard amount of blank calibration particles (ProSciTech) as determined using a hemocytometer.

For flow cytometric analysis of cell death, BAL cells were incubated with AF647-conjugated Annexin V (BioLegend) and 5 µg/ml propidium iodide (PI; Thermo Fisher Scientific) resuspended in binding buffer (10 mM HEPES pH 7.4, 150 mM NaCl, and 2.5 mM CaCl_2_ buffer) and analyzed by flow cytometry using an Aurora flow cytometer (Cytek Biosciences) and FlowJo^TM^ 10 analysis software (BD Biosciences).

### Immunofluorescence and immunohistochemical staining of lung tissue sections

NBF-inflated and fixed lung tissues were submerged in NBF for 48 h before being embedded in paraffin wax. Longitudinal lung tissue sections (4 µm) mounted on histology slides were then dewaxed and rehydrated. Sections were then microwaved for 15 min (high power) and 5 min (low power) in EDTA buffer (1 mM EDTA, pH 8) for heat-induced antigen retrieval. Slides were then blocked with CAS-Block Histochemical Reagent (Thermo Fisher Scientific) for 1 h before incubation with a cocktail of primary antibodies: cleaved GSDME (38821S, Cell Signalling Technology), E-cadherin (AF648SP, R&D Systems), and CD45^+^ (clone 30-F11, BD Biosciences) at 4 °C overnight. Slides were then washed with PBS containing 0.01% Tween 20 and incubated with anti-goat (Abcam), anti-rabbit, or anti-rat (Thermo Fisher Scientific) secondary antibodies. Slides were then washed, stained with Hoechst 33342 nuclear staining (Thermo Fisher Scientific), and mounted using Fluorescence Mounting Medium (Agilent). Lung sections were examined and imaged at 20x magnification using a Nikon A1R confocal microscope (Nikon, Tokyo, Japan). Five random fields of view (FOV) were analyzed per mouse using HALO software (Indica Labs, New Mexico, USA). Four fluorescent colors were used in the image analysis. Individual cells were identified using the nuclear Hoechst signal, and the expression of cleaved GSDME, E-cadherin, and CD45 was analyzed. Parameter settings in the HighPlex FL algorithm were verified by manual visual inspection of positive cells from randomly selected images. The number and percentage of cells that were positive for cleaved GSDME and double positive for E-cadherin/cleaved GSDME and CD45/cleaved GSDME were quantified per FOV.

Terminal deoxynucleotidyl transferase-mediated dUDP nick-end labeling (TUNEL) assay was performed on lung tissue sections using the ApopTag Peroxidase In Situ Apoptosis Detection Kit (Merck Millipore), according to the manufacturer’s instructions. Lung sections were counterstained with hematoxylin and cover-slipped with D.P.X. mounting medium (Sigma Aldrich). Lung sections were viewed using an Olympus DP74 microscope (Olympus, Tokyo, Japan), and five random single-plane images per mouse section were imaged with an Olympus DP74 color camera using Olympus cellSens Dimension software at 10x magnification. ImageJ software (National Institutes of Health, USA) was used to quantify TUNEL labeling intensity (percentage positive pixel intensity per FOV), color deconvolution was performed, and a threshold was set on 3,3′-diaminobenzidine (DAB) intensity, which was applied to all lung sections.

### Assessment of lung damage and pathology

Longitudinal lung tissue sections (4 µm) were prepared and stained with hematoxylin and eosin (H&E) by the Monash University Histology Platform. Tissues were graded for alveolitis and peribronchial inflammation on a subjective scale of 0 to 5 (0 = no inflammation, 1 = very mild, 2 = mild, 3 = moderate, 4 = marked, and 5 = severe inflammation), as previously performed [16, 19]. Sections were also scored for features of epithelial damage such as the presence of debris in the airspace, epithelial denudation, and thickening of the epithelial wall (0 = no obvious damage, 1 = mild, 2 = moderate, 3 = marked, and 4 = severe). Sections were blinded and randomized, and samples corresponding to the least severe and most severe were assigned scores of 0 and 4/5, respectively, and five random fields per mouse were graded by three independent researchers. Lung sections were viewed on an Olympus BX60 microscope and photographed at 10x magnification with an Olympus DP74 color camera using Olympus cellSens Dimension software.

As further assessment of lung damage, levels of adenosine triphosphate (ATP) and total protein levels in BAL fluid were determined, as previously described [19], using the Cell Titer Glo 2.0 Cell Viability Assay (Promega) and Pierce^TM^ BCA Protein Assay Kit (Thermo Fisher Scientific), respectively.

### Data and statistical analysis

Data is presented as mean ± standard deviation (SD). Data from each individual animal are shown. Sample sizes were used based on previous extensive experience in the laboratory within similar studies. The investigator was blinded to the group allocation for the assessment of histology but was not blinded for other experiments. Data were tested for normality and analyzed by GraphPad Prism Version 9 software (Graphstats Technologies, Bangalore, India). A Student’s *t* test (two-tailed unpaired) was used when comparing two values. When comparing three or more sets of values, a one-way or two-way analysis of variance (ANOVA) was used with either Tukey’s or Dunnett’s multiple comparisons post-hoc test. Survival proportions were compared using the Mantel–Cox log-rank test. A *P* value < 0.05 was considered statistically significant.

## Results

### IAV infection induces cleavage of GSDME in human bronchial epithelial cells

**Fig. 1:**
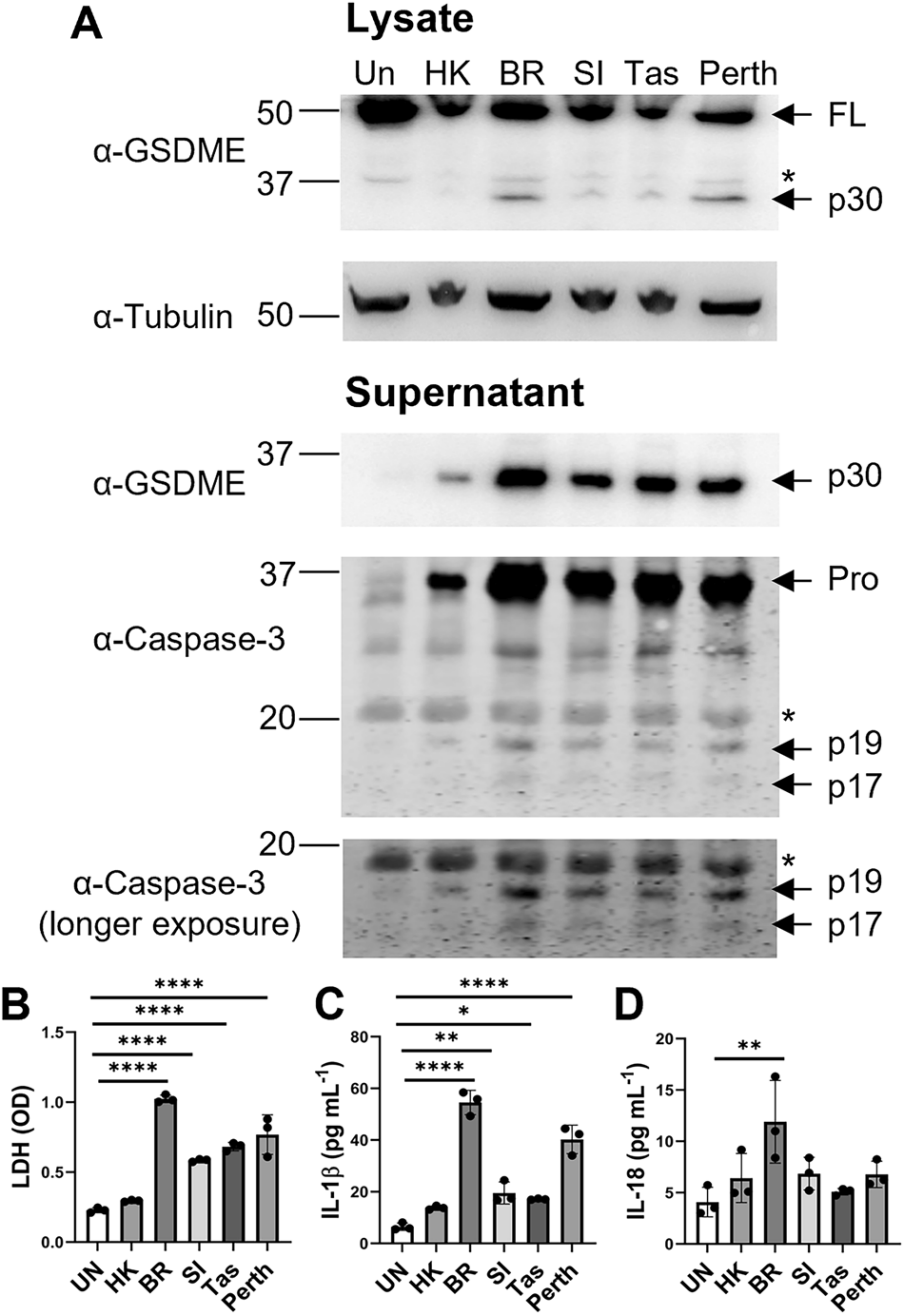
IAV infection promotes GSDME cleavage in human bronchial epithelial cells. Human normal bronchial epithelial HBEC3-KT cells were infected with human IAV HKx31 (HK; H3N2), Brazil/78 (BR; H1N1), Solomon Islands/06 (SI; H1N1), Tasmania/09 (Tas; pandemic H1N1), or Perth/09 (Perth; H3N2) at a multiplicity of infection of 3 for 24 h. Uninfected (UN) cells were included for comparison. **A** Immunoblot of GSDME, caspase-3, and tubulin protein in cell lysates (top) and cell supernatants (bottom). Arrows indicate full length (FL) and cleaved N-terminal p30 subunit of GSDME, as well as caspase-3 precursor (pro) and cleaved p17 and p19 subunits. * Indicates a non-specific band. Data are representative of three independent experiments. Levels of **B** LDH (OD; optical density), **C** IL-1β, and **D** IL-18 in cell supernatants, determined by colorimetric assay or ELISA. **B**–**D** Data are shown as experimental replicates that are representative of three independent experiments. Data are expressed as mean ± SD. *P < 0.05, **P < 0.01, ****P < 0.0001, one-way ANOVA.

The pyroptotic effector GSDME can be cleaved by apoptotic caspase-3, releasing its active NT domain [12, 13]. Here, we sought to confirm the GSDME cleavage response in epithelial cells, the primary target of IAV. Full-length GSDME was observed in uninfected cell lysates, suggesting that GSDME is constitutively expressed in human bronchial epithelial cells (Fig. 1A). At 24 h following H1N1 and H3N2 infection, expression of the active p30 N-terminal subunit of GSDME increased in cell lysates and supernatants at varying amounts, particularly with Brazil/78. Notably, the magnitude of GSDME activation induced by different IAV strains correlated with the expression of the active p17 and p19 subunits of caspase-3 (Fig. 1A). Additionally, akin to our past work on GSDMD [16], levels of GSDME cleavage also positively correlated with heightened levels of LDH and IL-1β in cell supernatants (Fig. 1B, C). Low levels of IL-18 release were exclusively observed in cell supernatants following Brazil/78 infection (~2-fold; Fig. 1D). Together, these data demonstrate that H1N1 and H3N2 IAVs vary in their ability to promote the activation of GSDME and pyroptotic events in human bronchial epithelial cells.

### GSDME and GSDMD are cooperatively active in human bronchial epithelial cells following IAV infection

**Fig. 2:**
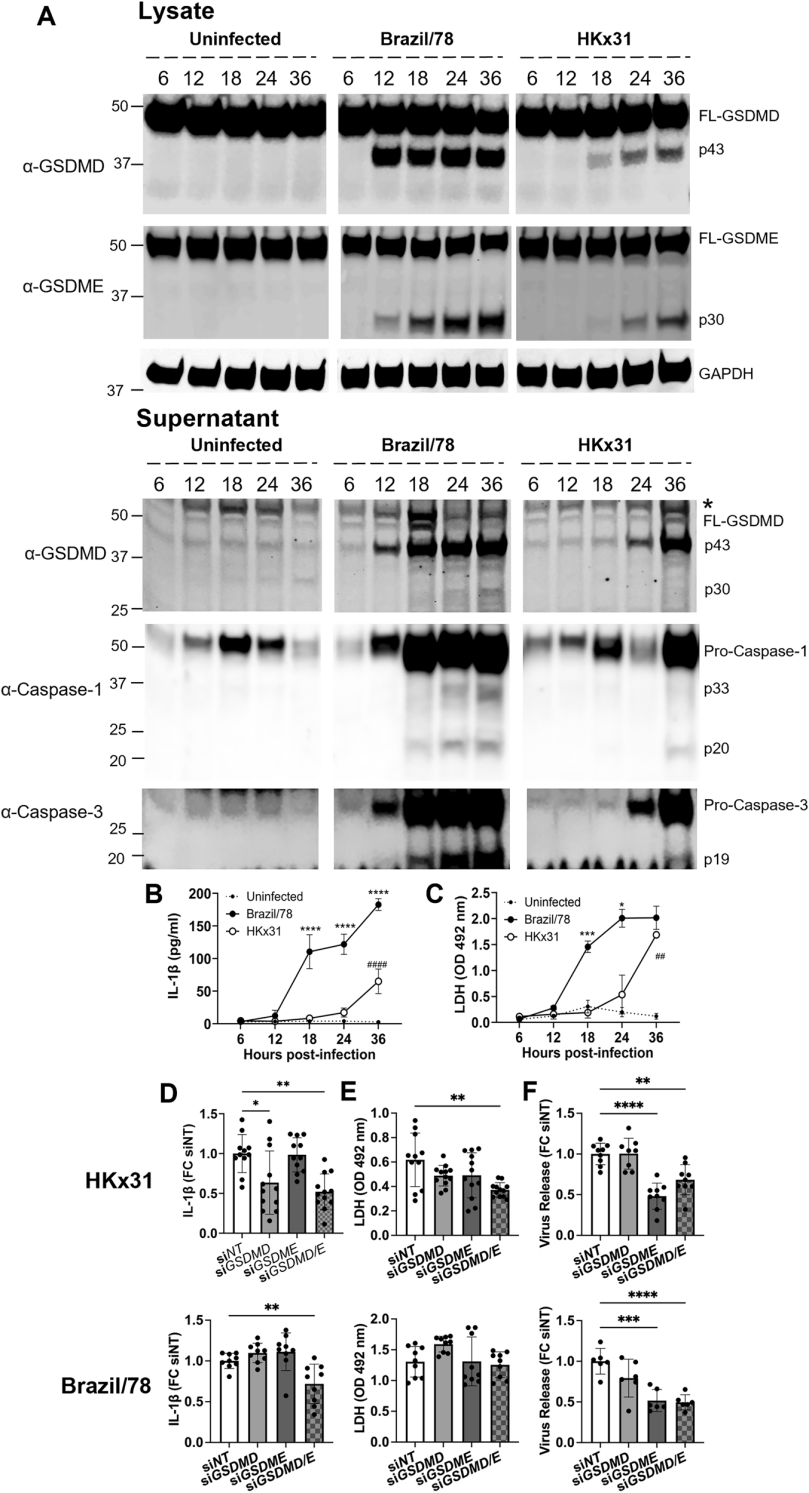
GSDME and GSDMD are active in human bronchial epithelial cells following IAV infection. **A**–**C** Human normal bronchial epithelial HBEC3-KT cells were infected with human IAVs HKx31 (H3N2) or Brazil/78 (H1N1) at a multiplicity of infection of 3. Uninfected cells were included for comparison. **A** Immunoblot of GSDMD, GSDME, caspase-1, caspase-3, and GAPDH protein in cell lysates (top) or supernatants (bottom) at 6-36 h post-infection. Full-length (FL) and cleaved subunits of GSDME (active p30) and GSDMD (inactive p43 and active p30), as well as precursor (pro) and cleaved subunits of caspase-1 (p33, p20) and caspase-3 (p19), are shown. * Indicates a non-specific band. Data are representative of three independent experiments. Levels of **B** IL-1β and **C** LDH (OD; optical density) in cell supernatants, determined by ELISA or colorimetric assay, respectively. **B**, **C** Data presented as mean ± SD. *P < 0.05, **P < 0.01, ***P < 0.001, ****P < 0.0001, Brazil/78 vs HKx31, two-way ANOVA. ##*P* < 0.01, ####*P* < 0.0001, uninfected vs HKx31, two-way ANOVA. Data are representative of three independent experiments pooled. **D**–**F** siRNA (si) silencing of GSDMD (si*GSDMD*), GSDME (si*GSDME*), or GSDMD/E (si*GSDMD/E*) expression in HBEC3-KT cells. Non-targeting (NT) siRNA control was included. At 48 h post-transfection, cells were infected with HKx31 or Brazil/78 at a multiplicity of infection of 3. **D** IL-1β levels and **E** LDH release (OD) in cell supernatants at 24 h post-infection, determined by ELISA or colorimetric assay, respectively. Data pooled from three or four independent experiments. For normalization between experiments, IL-1β data is presented as a fold change to NT control ± SD. *P < 0.05, **P < 0.01, one-way ANOVA. **F** Infectious virus in cell supernatants at 24 h post-infection, determined by plaque assay. Data pooled from two or three independent experiments and presented as fold change to NT control ± SD to normalize experimental variation. **P < 0.01, ***P < 0.001, ****P < 0.0001, one-way ANOVA.

We have previously shown that infection of human bronchial epithelial cells with IAV results in GSDMD cleavage and activation [16]. Thus, having established IAV infection induces both GSDMD and GSDME activation, we next compared the kinetics of relevant cell death responses in human bronchial epithelial cells infected with IAV Brazil/78 (H1N1; induces strong pyroptotic response) or HKx31 (H3N2; induces weak pyroptotic response). Namely, GSDMD, GSDME, caspase-1, and caspase-3 cleavage (Fig. 2A), as well as IL-1β (Fig. 2B) and LDH (Fig. 2C) release, were temporally examined. Cleaved GSDME was readily detected in cell lysates from 12 h post-Brazil/78 infection (Fig. 2A). In contrast, the response following HKx31 infection was delayed, with cleaved GSDME not detected until 24 h. Caspase-3-mediated inactivation of GSDMD was also rapidly observed following Brazil/78 and HKx31 infection, with the inactive p43 subunit strongly detected in cell lysates at 12 or 18 h, respectively. Activation of GSDME and inactivation of GSDMD closely correlated with the strength and kinetics of caspase-3 cleavage. GSDMD activation succeeded GSDME activation, with the p30 subunit of GSDMD, as well as caspase-1 cleavage, observed in the cell supernatants from 18 h or 36 h following Brazil/78 and HKx31 infection. Notably, the intensity of GSDME, GSDMD, caspase-3, and caspase-1 cleavage was strikingly more pronounced over the time course of Brazil/78 infection (Fig. 2A), correlating with significantly greater IL-1β (Fig. 2B) and LDH (Fig. 2C) release at 18 and 24 h post-infection. Lastly, IL-1β release temporally mirrored LDH release following both Brazil/78 and HKx31 infection.

We next performed siRNA targeting of the gasdermins in human bronchial epithelial cells to ascertain the individual roles for GSDMD and GSDME in IAV infection. Suppression of GSDMD and/or GSDME expression was confirmed at 24 h following infection via immunoblot (Fig. S1). When GSDMD was suppressed, GSDME cleavage and activation remained largely constant following Brazil/78 and HKx31 infection compared to the non-targeting (NT) control (Fig. S1). In contrast, siRNA knockdown of GSDME appeared to enhance GSDMD inactivation (p43 subunit) in response to both strains. Consistent with our earlier findings (Fig. 2A), the active p30 subunit of GSDMD was not detectable in cell lysates at 24 h (Fig. S1). Importantly, analysis of IAV responses at 24 h revealed that knockdown of GSDMD expression was sufficient to limit IL-1β secretion following HKx31 infection (Fig. 2D). However, dual silencing of GSDMD and GSDME was required to reduce IL-1β secretion following Brazil/78 infection (Fig. 2D). Similarly, LDH release was only significantly compromised following HKx31 infection when both GSDMD and GSDME were suppressed (Fig. 2E). In contrast, at the peak of LDH release observed upon Brazil/78 infection, GSDMD, GSDME, or GSDMD/E suppression failed to impact cell lysis, suggesting a further mechanism exists to compromise membrane integrity. Lastly, targeting of GSDME alone, or in combination with GSDMD, significantly limited infectious virus release at 24 h following both Brazil/78 and HKx31 infection (Fig. 2F).

In summary, these data demonstrate that IAV infections, albeit with a differing magnitude and kinetics, dominantly promote caspase-3-mediated GSDME activation rather than inflammasome-associated caspase-1 and GSDMD activation in human bronchial epithelial cells. We also show that despite this temporal difference in activation, suppression of both GSDMD and GSDME is required to significantly dampen both cell lysis and inflammation following Brazil/78 infection, suggesting possible redundancy. However, impressively, we find that loss of GSDME alone is sufficient to limit infectious virus release.

### IAV infection induces cleavage of GSDME in vivo

**Fig. 3:**
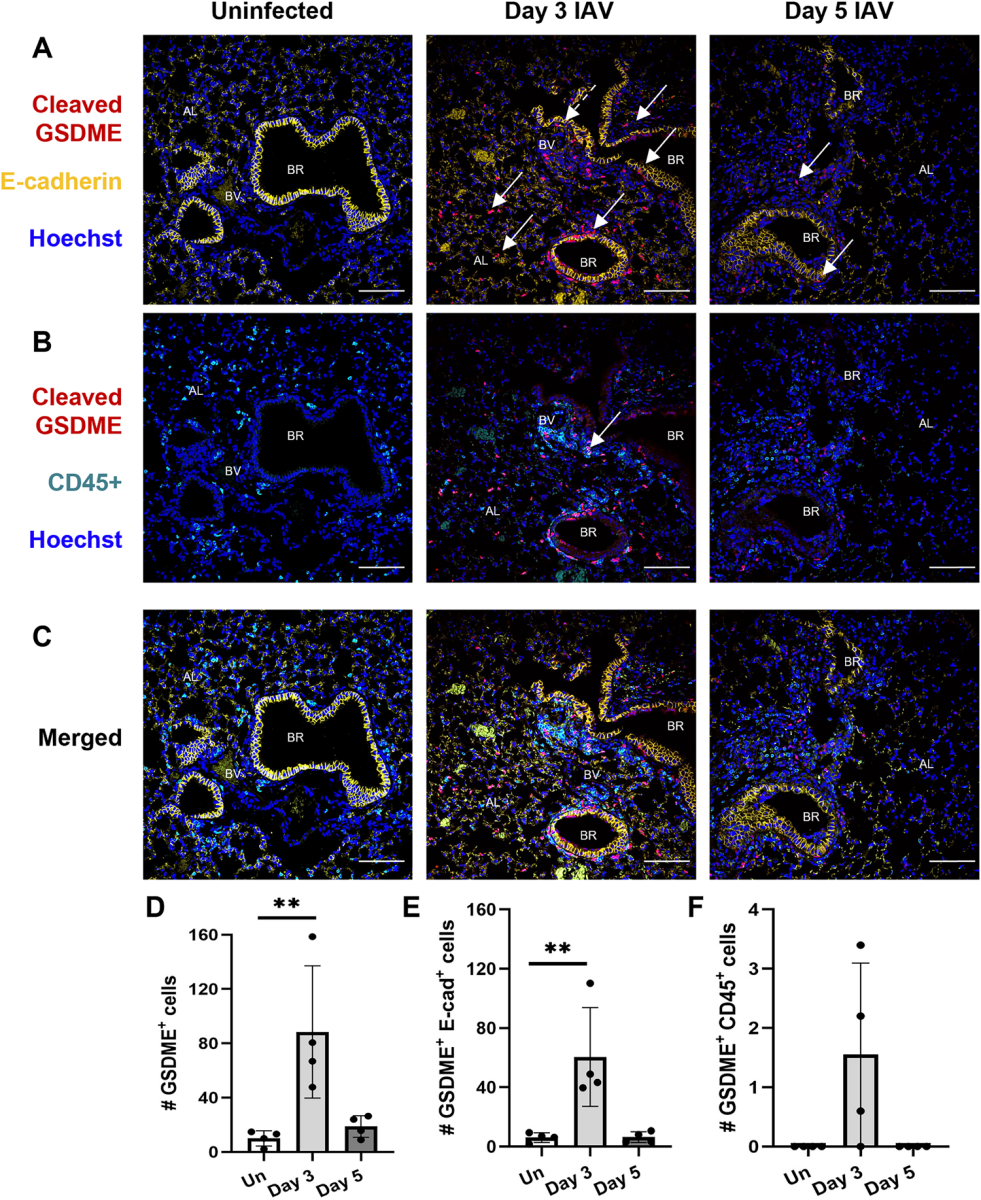
IAV infection promotes cleavage of GSDME in vivo in murine lung epithelial cells*.* Wildtype mice were infected with 10^4^ PFU of HKx31 IAV, and lung tissues were formalin inflated and fixed on days 3 and 5 post-infection. Uninfected (Un) controls were included for comparison. n = 4 per group. **A**–**C** Expression of cleaved GSDME (red) in lung tissue sections measured by confocal microscopy. Hoechst nuclear stain is shown in blue. Co-staining for E-cadherin (yellow; epithelial cells) and CD45 (cyan; leukocytes) was also performed. Representative images at 40x magnification (scale bar 100 µm) showing colocalization of **A** cleaved GSDME with E-cadherin and **B** cleaved GSDME with CD45. (C) Merged images showing cleaved GSDME, E-cadherin, CD45, and Hoechst. **A**–**C** Bronchiole (BR), alveolus (AL), and blood vessels (BV) are labeled. Solid arrows indicate examples of colocalization. Dashed arrows indicate cleaved GSDME in cells around blood vessels. Number (#) of **D** cleaved GSDME^+^, **E** E-cadherin (E-cad)^+^ and cleaved GSDME^+^, and **F** CD45^+^ and cleaved GSDME^+^ cells per field of view (FOV). **D**–**F** Five random FOVs were analyzed per animal using HALO software. Data are presented as mean cell count ± SD, with each data point representing an individual animal. P** < 0.01, one-way ANOVA.

We have previously shown by confocal imaging of lung tissue sections that severe HKx31 H3N2 IAV infection induces cleavage of GSDMD in vivo and primarily in E-cadherin^+^ epithelial cells lining the bronchioles [16]. In this study, confocal imaging of cleaved GSDME expression in lung tissue sections from uninfected and infected wildtype mice was performed (Fig. 3 and Fig. S2). The percentage of cells that were positive for cleaved GSDME significantly increased at day 3 post-infection (Fig. S2F), correlating with a > 8-fold increase in GSDME^+^ cell counts (Fig. 3D). At day 5, cleaved GSDME^+^ cell counts were similar to uninfected controls. Cleaved GSDME expression overlapped with E-cadherin^+^ epithelial cells in the bronchioles and alveoli (Fig. 3A). At day 3, the number of cells that were double positive for E-cadherin and cleaved GSDME (Fig. 3E; ~60 cells per FOV) was ~40-fold higher than the number of cells that were double positive for CD45 and cleaved GSDME (Fig. 3F; ~1-2 cells per FOV). Cleaved GSDME was also observed in E-cadherin^-^ and CD45^-^ cells, including endothelial cells lining the blood vessels (Fig. 3C–F). As expected, cleaved GSDME^+^ cells were not detected in lung sections from IAV-infected *Gsdme*^−/−^ mice (Fig. S2B, F). Altogether, IAV infection induced activation of GSDME in vivo in alveolar and bronchial epithelial cells.

### Mice lacking GSDME are more resistant to severe IAV infection

**Fig. 4:**
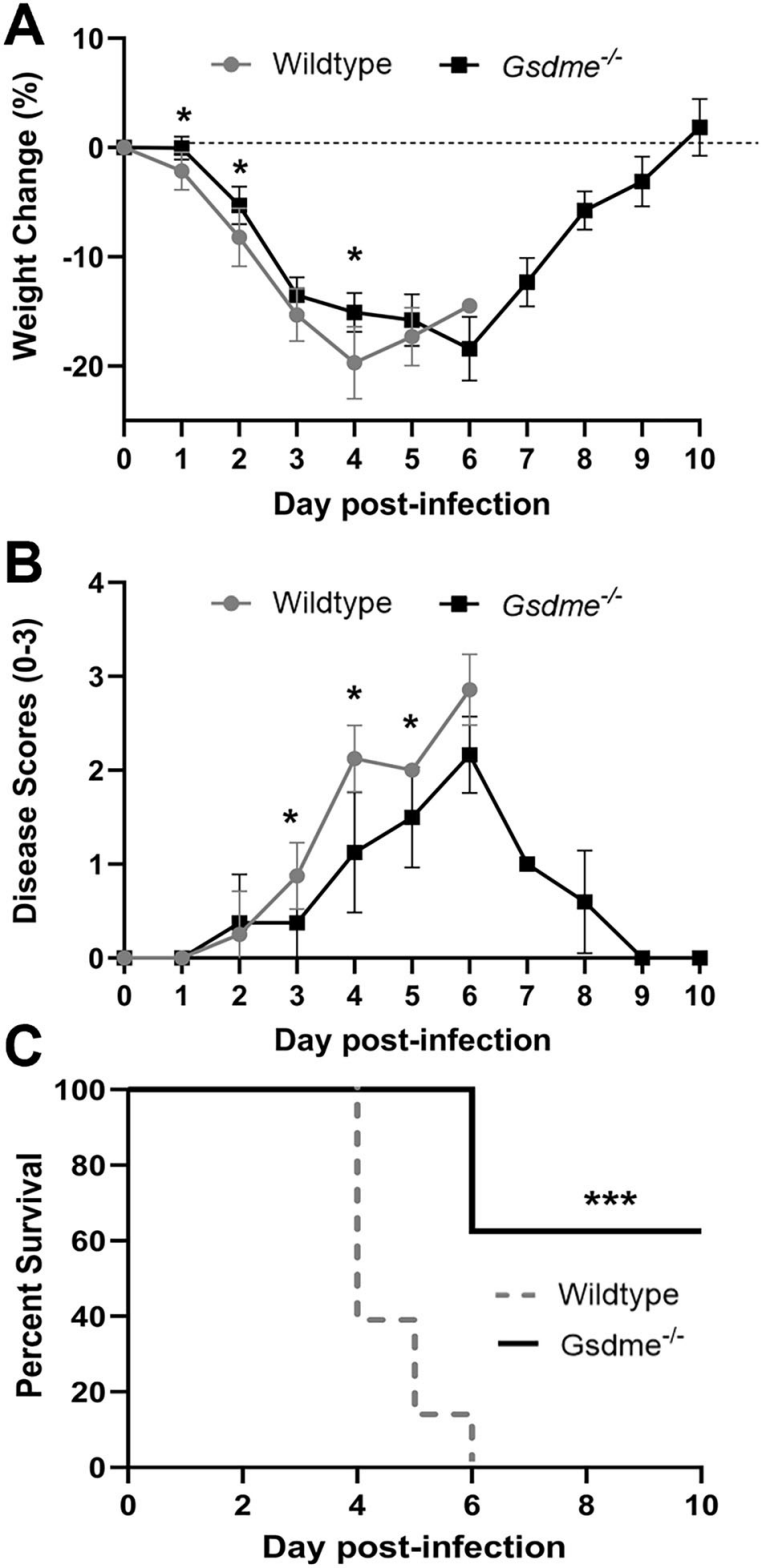
GSDME deficiency limits the severity of IAV infection. *Gsdme*^−/−^ mice and wildtype littermate controls were infected with 10^4^ PFU of HKx31 (H3N2) as a model of severe IAV infection. n = 8 per group. **A** Mouse weights were recorded daily and presented as mean percent weight change ± SD. *P < 0.05, two-tailed, unpaired Student’s *t* test. **B** Clinical signs of disease on a scale of 0–3 were scored daily, as described in the methods. *P < 0.05, two-tailed, unpaired Student’s *t* test. Results are expressed as mean ± SD. **C** Survival curves as shown. ***P < 0.001, Mantel–Cox log-rank test.

Given that IAV infection induced GSDME cleavage in the lung in vivo, we examined a potential role for GSDME in modulating clinical manifestations using a model of severe IAV infection. *Gsdme*^*−/−*^ mice and wildtype littermates were infected with 10^4^ PFU of HKx31 H3N2 IAV, and weight loss and clinical signs of disease (on a scale of 0–3, as described in the Methods) were monitored for up to 10 days (Fig. 4). Mice that lost ≥20% of their original body weight or displayed a clinical disease score of 3 were euthanized as per ethical requirements. Interestingly, *Gsdme*^*−/−*^ mice displayed a slower rate of weight loss until day 4 post-infection compared to their wildtype littermate controls (Fig. 4A). Further, *Gsdme*^*−/−*^ mice exhibited reduced clinical disease scores on days 3 to 5 (Fig. 4B). By day 6 post-IAV infection, all wildtype mice required euthanization (Fig. 4C). Conversely, 5 out of 8 *Gsdme*^*−/−*^ mice survived and returned to their original body weight by day 10 (Fig. 4A, C). These findings imply that GSDME contributes to severe IAV disease severity.

### Absence of GSDME limits neutrophil infiltration into the airway during IAV infection

**Fig. 5:**
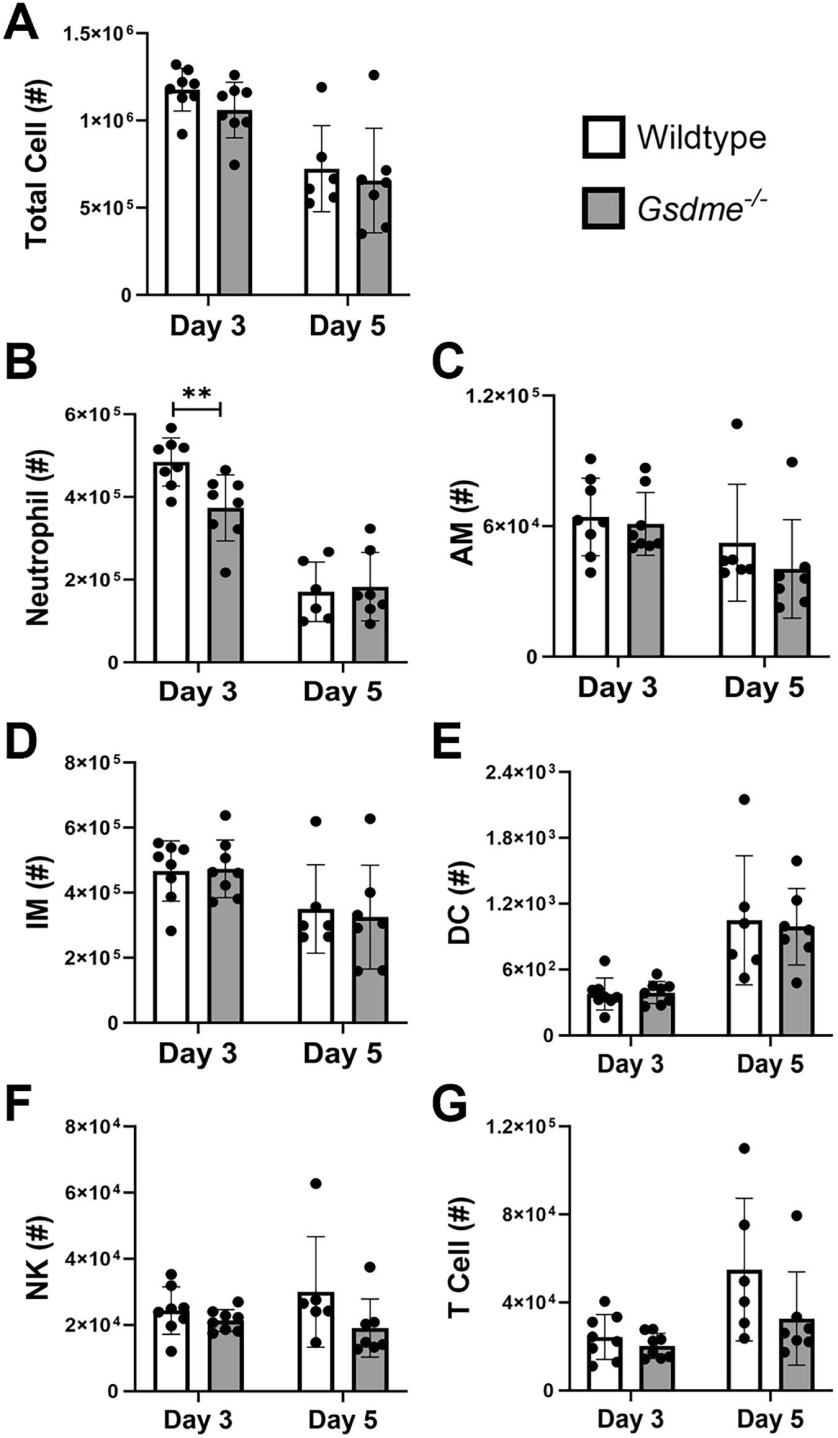
GSDME deficiency limits neutrophil infiltration in the airways. Wildtype and *Gsdme*^−/−^ mice were infected with 10^4^ PFU HKx31 IAV, and BAL was performed on days 3 and 5 post-infection. Numbers (#) of **A** total live cells, **B** neutrophils, **C** alveolar macrophages (AMs), **D** Ly6C^+^ inflammatory monocytes/macrophages (IMs), **E** dendritic cells (DC), **F** natural killer (NK) cells, and **G** T cells in the BAL, determined by flow cytometry. **A**–**G** *n* = 6, 7, or 8 per group. Data are presented as mean ± SD, with each data point representing an individual animal. **P < 0.01, two-tailed, unpaired Student’s *t* test.

IAV infection elicits immune cell infiltration into the lung that is characteristic of disease severity. We next investigated if GSDME influences immune cell recruitment into the airway at days 3 and 5 post-infection (Fig. 5). Cells collected by bronchoalveolar lavage (BAL) of infected wildtype and *Gsdme*^−/−^ mice were analyzed by flow cytometry. Total airway cellularity and numbers of infiltrating neutrophils, natural killer (NK) cells, Ly6C^hi^ inflammatory monocytes/macrophages (IMs), dendritic cells (DCs), and T cells, as well as resident alveolar macrophages (AMs), were determined (Fig. S3A). At day 3 post-infection, neutrophil numbers in the airway of *Gsdme*^*−/−*^ mice were significantly reduced compared to their wildtype littermate controls (Fig. 5B), despite no difference being observed in total cell counts (Fig. 5A). No significant changes to the numbers of AMs, IMs, DCs, NK cells, and T cells in the BAL were found between the two genotypes at either time point (Fig. 5C–G).

We then investigated alterations in leukocyte cell death using Annexin V and PI staining at day 3 post-infection. No significant differences in the frequency of dead (Annexin V^+^ PI^+^) or dying (Annexin V^+^ PI^-^) neutrophils were observed (Fig. S3B). Further, there were no differences in the cell surface expression of functional markers major histocompatibility complex (MHC) class II and Siglec-F on neutrophils (Fig. S3E,F), as well as their granularity (as indicated by their side scatter (SSC) characteristics; Fig. S3G), suggesting GSDME is dispensable for the activation of neutrophils. Frequencies of dead and dying AMs, which are resident cells that are susceptible to IAV infection [20], were also unchanged (Fig. S3C). Interestingly, despite *Gsdme*^−/−^ mice displaying equivalent numbers of infiltrating IMs at day 3 (Fig. 5D), there was a significant reduction in the frequency of dead (Annexin V^+^ PI^+^) IMs (Fig. S3D). Taken together, these results suggest GSDME deficiency limits neutrophil recruitment in the airway during severe IAV infection.

### GSDME deficiency limits local and systemic cytokine levels during IAV infection

**Fig. 6:**
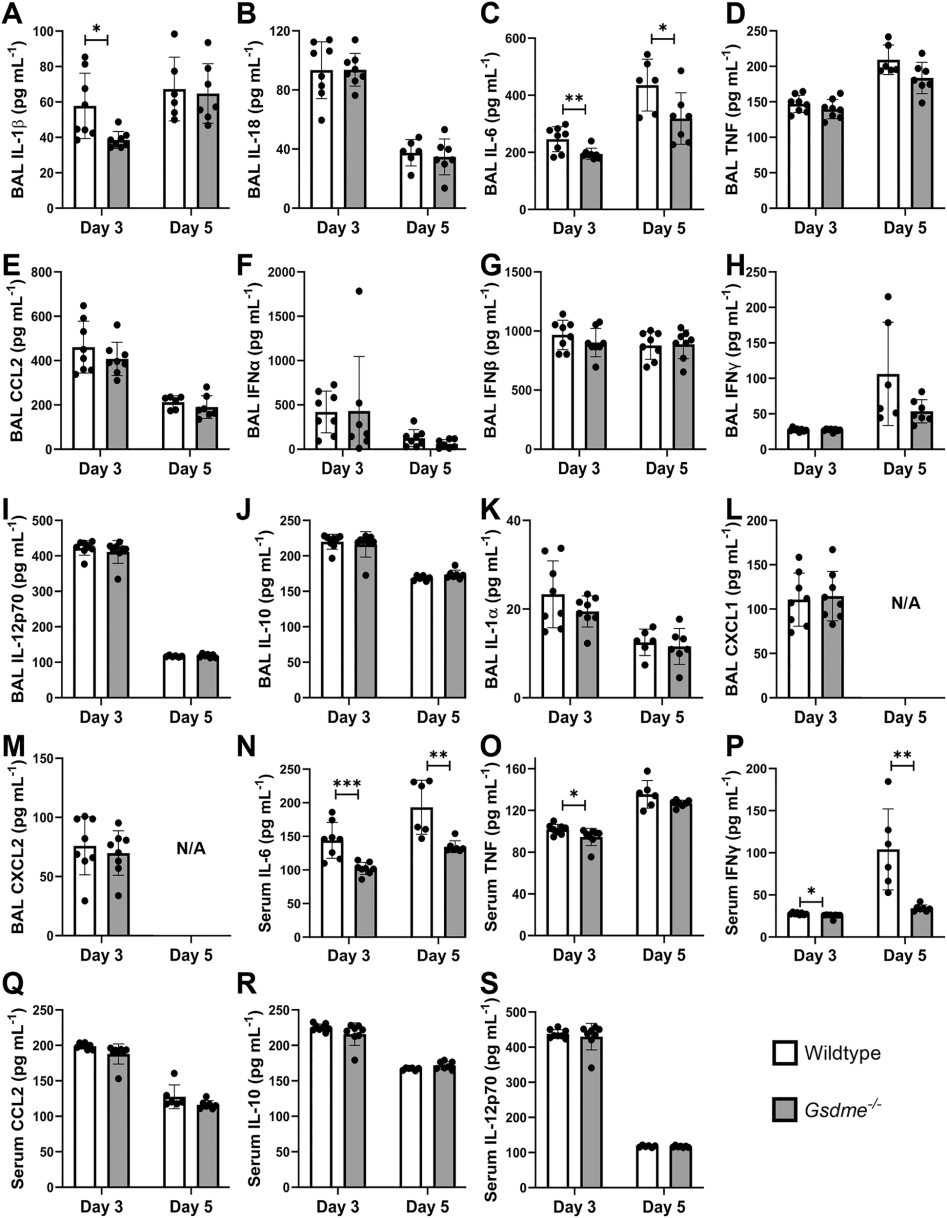
GSDME deficiency limits the production of local and systemic pro-inflammatory cytokines. *Gsdme*^−/−^ mice and wildtype littermates were infected with 10^4^ PFU HKx31 IAV. BAL (A-M) and serum (N-S) were collected on days 3 and 5 post-infection. Levels of **A** IL-1β, **B** IL-18, **C** IL-6, **D** TNF, **E** CCL2, **F** IFNɑ, **G** IFNβ, **H** IFNγ, **I** IL-12p70, **J** IL-10, **K** IL-1ɑ, **L** CXCL1, and **M** CXCL2 in BAL fluid. N/A indicates not assayed. Levels of **N** IL-6, **O** TNF, **P** IFNγ, (**Q**) CCL2, (**R**) IL-10, and (S) IL-12p70 in serum. **A**–**S** Data determined by cytokine bead array, or ELISA. *n* = 6, 7, or 8 per group. Data are presented as mean ± SD, with each data point representing an individual animal. *P < 0.05, **P < 0.01, ***P < 0.001, two-tailed, unpaired Student’s *t* test.

Cytokine storm, or hypercytokinemia, is considered a major characteristic and key driver of disease severity during IAV infection [3]. Given that *Gsdme*^*−/−*^ mice had improved disease resistance (Fig. 4), we examined levels of cytokines commonly associated with inflammatory responses in the airways (Fig. 6A–M) and blood (Fig. 6N–S) at days 3 and 5 post-infection. A modest but significant reduction in IL-1β levels in the BAL was observed in *Gsdme*^−/−^ mice at day 3, but not day 5 post-infection (Fig. 6A). Additionally, levels of IL-6 in the BAL were significantly reduced in *Gsdme*^−/−^ mice at both day 3 and 5 (Fig. 6C). No significant differences were observed in local levels of the cytokines IL-18, TNF, CCL2, IFNα, IFNβ, IFNγ, IL-12p70, and IL-10 (Fig. 6B, D–J). Neutrophil chemoattractants IL-1α, CXCL1, and CXCL2 were also unchanged in the BAL (Fig. 6K–M). Furthermore, concentrations of IL-6, TNF, and IFNγ were significantly reduced in the serum of *Gsdme*^*−/−*^ mice compared to wildtype controls at days 3 and/or 5 (Fig. 6N–P). While systemic levels of CCL2, IL-10, or IL-12p70 remained unchanged (Fig. 6Q–S). Overall, these data demonstrate that GSDME deficiency limits local and systemic cytokine production during severe IAV infection.

### Deficiency of GSDME limits viral burden and lung pathology during severe IAV infection

**Fig. 7:**
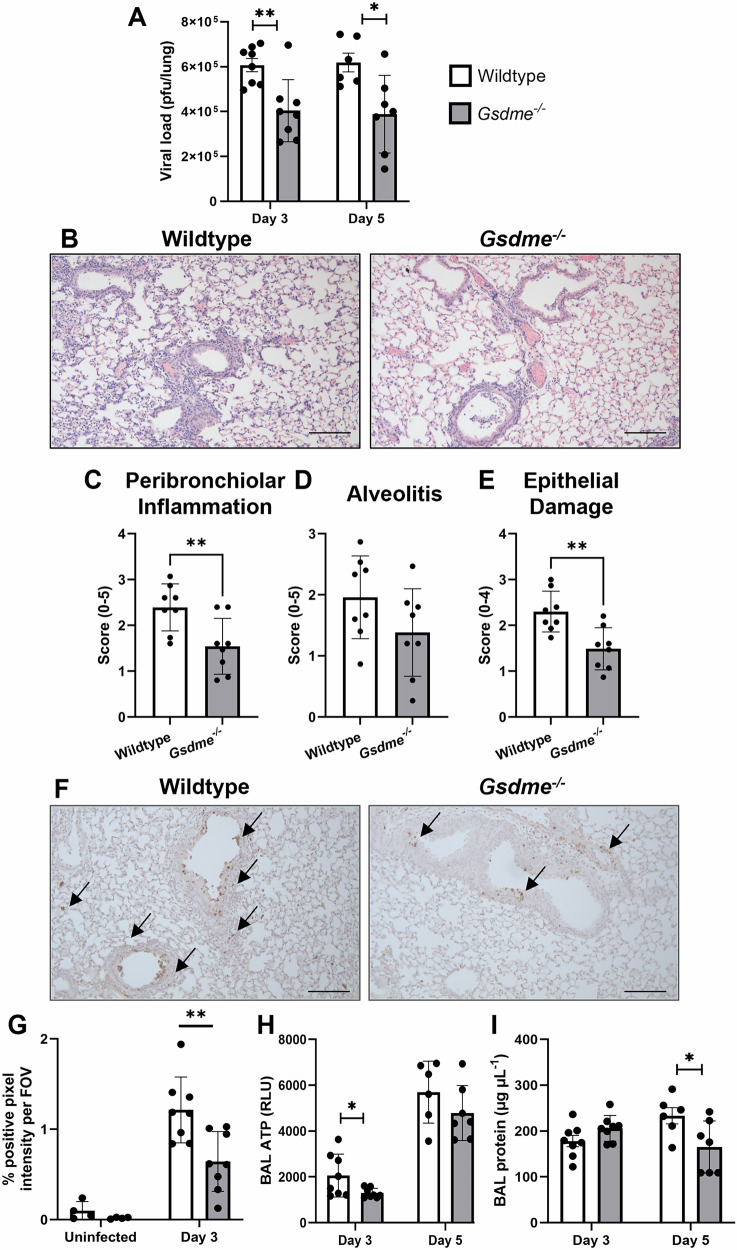
GSDME deficiency limits viral burden, lung damage, and cell death. *Gsdme*^−/−^ mice and wildtype littermates were infected with 10^4^ PFU HKx31 IAV. **A** Viral loads in lung tissues were measured by standard plaque assay at days 3 and 5 post-infection. Data are presented as plaque-forming units (pfu) per lung ± SD. *P < 0.05, **P < 0.01, two-tailed, unpaired Student’s *t* test. **B** Representa*t*ive images of H&E-stained lung tissue sections on day 3, imaged at 10x magnification. Scale bar 100 µm. Lung sections were randomized and scored blindly as described in the methods for **C** peribronchial inflammation, **D** alveolitis, and **E** epithelial damage. (C-E) Data are presented as mean ± SD, with each data point representing an individual animal. **P < 0.01, two-tailed, unpaired Student’s *t* test. **F** Representative images of TUNEL assay labeling of cell death in lung tissue sections at day 3 post-infection. **G** Quantification of TUNEL staining determined with ImageJ software. Uninfected controls were included for comparison. Five random fields of view (FOV) were analyzed per animal. Data are presented as the mean percentage positive pixel intensity per field of view (FOV). **H** Levels of ATP in BAL fluid at day 3 determined by luminescent assay (relative luminescence; RLU). **I** BAL total protein concentration determined by colorimetric assay at day 3. **G**–**I** n = 4, 6, 7, or 8 per group. Data are presented as mean ± SD, with each data point representing an individual animal. *P < 0.05, **P < 0.01, two-tailed, unpaired Student’s *t* test.

During severe IAV infection, excessive viral replication and inflammation can result in lung damage. Lung infectious viral burden (pfu/lung) was significantly reduced in GSDME-deficient mice at days 3 and 5 post-infection (Fig. 7A). Histological analysis of H&E-stained murine lung tissue sections revealed that the absence of GSDME significantly limits peribronchial inflammation and epithelial damage at day 3 post-infection (Fig. 7B, C, E). Correlating with attenuated lung damage in GSDME-deficient mice was a reduction in the levels of bronchial and alveolar epithelial cell death, as measured by TUNEL labeling of lung tissue sections (Fig. 7F–G). Moreover, the release of inflammatory cellular contents associated with pyroptosis, including the DAMP ATP, was also significantly reduced at day 3 compared to their wildtype controls (Fig. 7H). GSDME deficiency also led to reduced total protein levels in BAL fluids, which were significantly lower in *Gsdme*^*−/−*^ mice at day 5 (Fig. 7I), suggesting that GSDME contributes to the disruption of the lung epithelial barrier and increases permeability, allowing proteinaceous fluids to leak into the alveolar spaces. Collectively, these data demonstrate GSDME deficiency limits lung tissue damage, cell death, and viral loads during severe IAV infection.

## Discussion

Pyroptotic cell death is a lytic and inflammatory mode of death that is mediated by pore-forming gasdermin proteins. Gasdermin pores have been shown to facilitate the release of cytokines such as IL-1β [15]. Critically, the accumulation of these pores leads to plasma membrane rupture and subsequently the release of cellular contents, including inflammatory stimuli such as DAMPs and PAMPs, into the extracellular milieu. The gasdermin family member GSDME has been shown to be activated by caspase-3 during apoptosis, a form of cell death that has been well described to play a protective role during IAV infection [6]. In this study, we investigated the role of GSDME in a murine model of severe IAV. Here we demonstrated that GSDME is cleaved and activated in vivo in lung epithelial cells following IAV infection. Crucially, our in vivo studies reveal a detrimental role for GSDME in amplifying inflammation, lung pathology, and viral burden during severe IAV infection that led to worsened clinical signs and increased morbidity.

Epithelial cells are highly permissive to IAV, with infection typically leading to heightened viral burden and cell lysis [6, 21]. Here, we demonstrated that both H1N1 and H3N2 IAV subtypes promote cleavage of GSDME in normal human bronchial epithelial cells within 24 h post-infection and that this is strongly associated with terminal apoptotic caspase-3 activity (Fig. 1A). Consequently, we observed increased plasma membrane rupture and the release of the inflammasome-activated cytokines, IL-1β and IL-18 (Fig. 1B–D). These findings align with published observations where IAV infection of primary human alveolar and bronchial epithelial cells with avian H7N9 or the unusual neurotropic WSN H1N1 strain induced GSDME cleavage, plasma membrane rupture, and IL-1β release [17, 22], suggesting that GSDME-mediated pyroptosis is a form of cell death that is broadly elicited by different IAV strains. Interestingly, we have previously reported that IAV infection of human bronchial epithelial cells also results in cleavage of GSDMD, as well as the effector caspase-1 at 24 h post-infection [16].

Here, we extend these and our previous findings with GSDMD [16] and demonstrate that GSDME activation precedes GSDMD activation with greater intensity following IAV infection (Fig. 2A). We also show that the kinetics and magnitude of the responses differed between IAV strains, with Brazil/78 inducing earlier and more robust GSDME activation, plasma membrane rupture, and IL-1β release than HKx31 (Fig. 2B,C). As anticipated, the kinetics and magnitude of GSDME and GSDMD cleavage events observed correlated closely with the respective timing of caspase-3 and caspase-1 activation. In particular, we observed that early apoptotic caspase-3 activity limited GSDMD-mediated pyroptosis by cleaving GSDMD at D88 to produce an inactive p43 fragment and prevent NT-GSDMD p30 bioactive fragment generation [12].

Importantly, we confirmed, via siRNA targeting, that GSDMD and GSDME display functional overlap in human bronchial epithelial cells to promote LDH release (Fig. 2E). Although, ultimately, gasdermin pores appear to not be obligatory for LDH release, particularly to the strong pyroptotic trigger, Brazil/78, suggesting cell death plasticity exists and that other cell death pathways, like mixed lineage kinase domain-like (MLKL)-mediated necroptosis, may act in parallel or as a backup mechanism [23]. Largely consistent with our observations, Lee et al. recently reported that while human epithelial cell death induced by the mouse-adapted PR8 H1N1 strain was reduced by either caspase-1 or -3 inhibitor treatment, the greatest protection was only achieved upon pan-caspase inhibition [24].

Fitting with the notion that GSDMD and GSDME cooperatively regulate pyroptosis in human bronchial epithelial cells, suppression of both GSDMD and GSDME expression was also required to optimally attenuate IL-1β release in response to either IAV strain (Fig. 2D). Although it is important to note that GSDMD silencing also limited IL-1β release in response to HKx31 infection in a separable manner, suggesting that other lytic and non-lytic secretion mechanisms are in play in lung epithelial cells to promote damaging inflammation [25, 26]. Intriguingly, these results differ from recent studies that suggested that GSDMD pores do not act as a conduit for IL-1β secretion in IAV-infected mice [16], and our current findings in vivo, where we observed that the deletion of GSDME could modestly and selectively attenuate early IL-1β secretion in response to HKx31. This raises the possibility that there is functional divergency between species and/or that other cell types in the lung may utilize GSDME to facilitate IL-1β release.

Strikingly, silencing of GSDME in human bronchial epithelial cells limited infectious virus release following HKx31 and Brazil/78 infection (Fig. 2F). This is consistent with markedly reduced viral loads observed in *Gsdme*^*−/−*^ mice upon HKx31 infection (Fig. 7A). Future studies could elucidate if the impaired viral replication observed in GSDME-deficient mice is mechanistically a direct result of GSDME being required for the release of virus or a switch towards caspase-3-driven apoptosis and clearance of virus-containing apoptotic bodies [27]. In contrast to our findings, Guy et al. reported that silencing of GSDMD or GSDME in human bronchial epithelial cells enhanced replication, however, an atypical and highly virulent neurotropic WSN strain was utilized in their study [22]. Together, GSDME- and GSDMD-mediated pyroptosis is broadly and cooperatively induced in human bronchial epithelial cells by H1N1 and H3N2 IAV, albeit with differing kinetics and magnitudes, as well as pathological outcomes.

Our earlier report demonstrated that in vivo IAV infection induces cleavage of GSDMD, primarily in murine epithelial cells lining the bronchioles [16]. Here, confocal imaging of lung tissue sections from IAV-infected mice revealed cleaved GSDME in E-cadherin^+^ epithelial cells lining the bronchioles and alveoli at day 3 post-infection (Fig. 3A, E, and Fig. S2). The number (Fig. 3E) and proportion (Fig. S3G; ~3.22%) of cells that were double positive for E-cadherin and cleaved GSDME significantly increased at day 3. Interestingly, a lower number (Fig. 3F) and proportion (Fig. S3H; ~0.09%) of cells were double positive for CD45 and cleaved GSDME, suggesting that GSDME-mediated pyroptosis may be more predominantly active in epithelial cells in mice. Consistent with this, flow cytometric analysis of the proportion of dead and dying neutrophils, resident AMs, and infiltrating IMs revealed only a modest reduction in the frequency of Annexin V^+^ PI^+^ dead IMs in the absence of GSDME at day 5 (Fig. S3B–D), while TUNEL labeling of lung tissue sections revealed reduced bronchial and alveoli epithelial cell death (Fig. 7F, G). Of note, macrophages are susceptible to IAV infection, however, in contrast to epithelial cells, they do not support productive viral replication [21]. Collectively, these results suggest GSDME is active in bronchial and alveolar epithelial cells in vivo following IAV infection. Future studies could seek to further define the cell-specific contribution of gasdermins in myeloid and epithelial cells in vivo using conditional knockout mice.

In vitro studies have established that GSDMD and GSDME pores facilitate the release of inflammasome-dependent cytokines [15, 28]. Interestingly, in our previous study, mice lacking GSDMD displayed comparable levels of IL-1β and IL-18 [16]. Here, GSDME deficiency modestly reduced levels of IL-1β but not IL-18 in the airways following HKx31 infection (Fig. 6A, B), potentially due to differential regulation of the expression of these cytokines in vivo [29]. Consistent with these results, significant release of IL-1β but not IL-18 was observed following infection of human bronchial epithelial cells with several IAVs (Fig. 1D). While our in vivo results contrast those obtained by Wan et al., in which avian H7N9 IAV infection of *Gsdme*^*−/−*^ mice reduced levels of IL-1β and IL-18 in the lung at day 6 post-infection, we suspect that at this late time point, lung damage would be extensive. Furthermore, the multiplex array used by Wan and colleagues is unable to distinguish between precursor and active forms of these cytokines [17]. Importantly, we have previously shown that inhibition of the NLRP3 inflammasome or direct neutralization of IL-1β in vivo limits IAV disease severity [29–31]. IL-1β drives damaging inflammation by engaging the IL-1 receptor, leading to subsequent activation of the NF-κB pathway [32] and the production of pro-inflammatory cytokines such as IL-6, as well as IL-1β itself, promoting a feed-forward inflammation loop. In line with this, GSDME deficiency reduced levels of IL-6 locally in the airways and diminished systemic IL-6, TNF, and IFNγ levels in the serum of *Gsdme*^*−/−*^ mice (Fig. 6C, N-P). Interestingly, we observed cleaved GSDME in endothelial cells at day 3 post-infection (Fig. 3C), suggesting that GSDME could potentially promote endothelial damage and vascular leak of cytokines and other factors into the blood. However, further studies are needed to elucidate a potential role for GSDME in endothelial cells. Altogether, there is indeed a role of GSDME in amplifying levels of local and systemic pro-inflammatory cytokines.

The infiltration of immune cells into the airways is crucial to clearing the virus and restoring tissue homeostasis, however, responses need to be tightly controlled to limit damage to the epithelium. Neutrophils are one of the first immune cells to transmigrate into the IAV-infected lung, playing both protective and detrimental roles [33]. In this study, GSDME deficiency limited early neutrophil infiltration in the lung following IAV infection (Fig. 5B). This difference was not associated with perturbed neutrophil cell death in *Gsdme*^*−/−*^ mice (Fig. S3B), nor was it linked to impaired levels of neutrophil chemoattractants IL-1α, CXCL1, and CXCL2 (Fig. 6K–M), as we previously observed in the absence of GSDMD [16]. Instead, reduced neutrophil recruitment correlated with reduced levels of IL-1β and IL-6 in the airways (Fig. 6A, C), which have been shown to mediate neutrophil infiltration [29, 34]. Therefore, it appears GSDMD and GSDME activity may potentially promote neutrophil infiltration into the airways, potentially via different mechanisms.

Severe IAV infections can lead to the development of the life-threatening condition acute respiratory distress syndrome (ARDS). Compromised lung barrier integrity and buildup of proteinaceous fluid in the airways as a result of IAV-induced hyperinflammation and extensive epithelial cell damage or death are key features of ARDS [35]. In this study, GSDME deficiency reduced BAL total protein levels at day 3 post-IAV infection (Fig. 7I), indicative of reduced vascular leak and pulmonary edema. This correlated with a reduction in viral burden as well as lung pathology, including peribronchial inflammation and epithelial damage in *Gsdme*^*−/−*^ mice (Fig. 7A, C, E). Our findings partially complement a recent report by Wan et al. that found *Gsdme*^−/−^ mice displayed improved survival outcomes and lung pathology following avian H7N9 infection that was associated with reduced lung viral burden at day 6, but not at day 3 post-H7N9 infection [17]. However, in our study, we observed a reduction in viral burden at both day 3 and day 5 post-infection with HKx31 IAV (Fig. 7A), which may reflect differing infection kinetics between IAV strains. Critically, we also observed reduced epithelial damage and cell death in the absence of GSDME that correlated with lower levels of the danger signal ATP in the airways (Fig. 7H). ATP is released by damaged or dying cells to perpetuate inflammation and has recently been demonstrated to boost neutrophil recruitment into the lungs and promote lung damage during IAV infection [36]. Consequently, ATP release in GSDME-deficient airways may address the reduced neutrophil levels in the lung we observed.

In this study, we have demonstrated an adverse role for GSDME in aggravating inflammation and lung pathology during severe IAV infection. Specifically, the absence of activated GSDME largely in lung epithelial cells following IAV infection of mice resulted in *Gsdme*^*−/−*^ mice exhibiting reduced local and systemic inflammation and viral burden and improved lung pathology. There is an urgent unmet need for an effective host-targeted therapy for respiratory virus infections, particularly for a pathogen such as IAV, which is capable of rapid mutation, and when existing antiviral drugs are mostly ineffective. Given that hyperinflammation in the lungs resulting from a dysregulated host immune response is a major contributor to adverse clinical outcomes, inhibiting the GSDMD and/or GSDME pathways may be a potential therapeutic strategy that limits the progression and development of severe IAV-induced lung damage. Importantly, the development of targeted gasdermin inhibitors in the future would allow assessment of such a strategy in dampening pyroptotic cell death during severe IAV infection.

## Supplementary information

Supplemental Material

## Data Availability

All datasets generated and analyzed during this study are included in this published article and its Supplementary Information files. Full-length uncropped western blots are available in the Supplemental material files. Additional data are available from the corresponding author on reasonable request.
