# Supplementary material for: Gasdermin E deficiency limits inflammation and lung damage during influenza virus infection
Source: Cell Death Dis. 2025 Jun 6;16(1):440. doi: 10.1038/s41419-025-07748-0 (PMC12144252; doi:10.1038/s41419-025-07748-0)

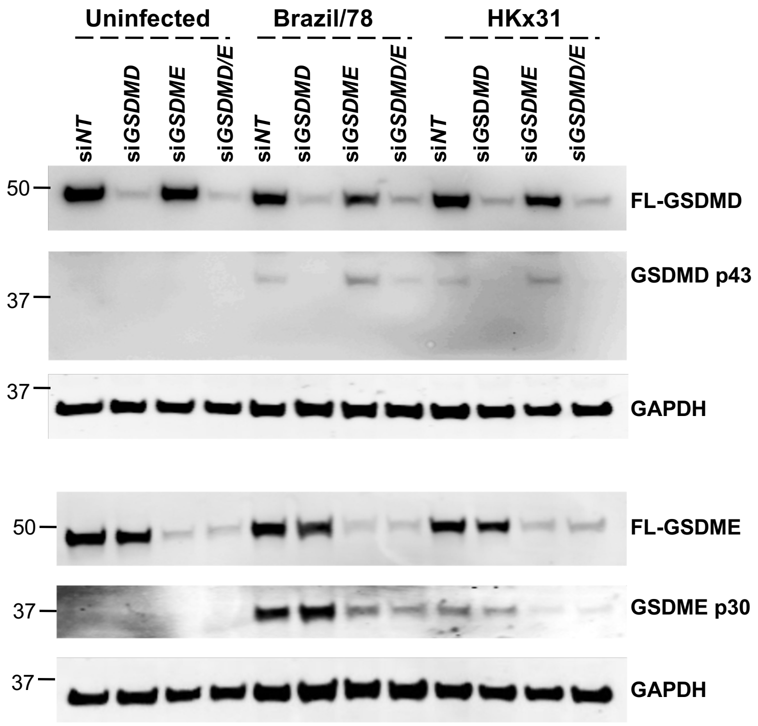

Figure S1. Suppression of gasdermin expression in human bronchial epithelial cells. siRNA (si) silencing of GSDMD (siGSDMD), GSDME (siGSDME), or GSDMD/E (siGSDMD/E) expression in HBEC3-KT cells was performed. Non-targeting (NT) siRNA control was included. At 48 h post-transfection, cells were infected with Brazil/78 (H1N1) or HKx31 (H3N2) at a multiplicity of infection of 3. Uninfected cells were included for comparison. Immunoblot of GSDMD, GSDME, and GAPDH protein in cell lysate at 24 h post-infection. Full-length (FL) and cleaved subunits of GSDMD (inactive p43) and GSDME (active p30) are shown. Data are representative of pooled triplicate wells and two independent experiments.

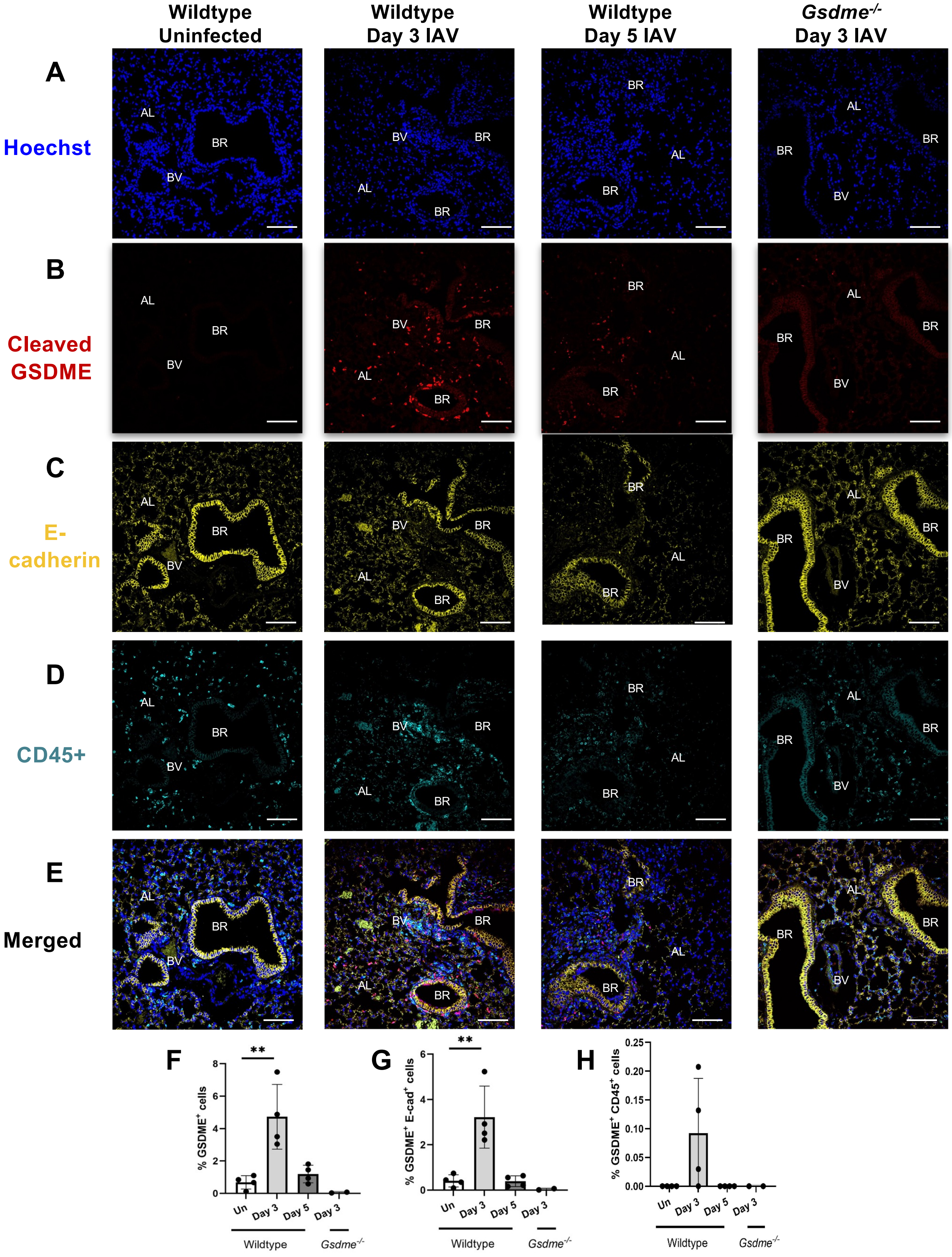

Figure S2. Cleaved GSDME expression in lung tissue sections. (A-E) *Gsdme*$^{-/-}$ mice and wildtype littermates were infected with 10^4 PFU of HKx31. n=4 per group. Confocal imaging of lung tissue sections on days 3 and 5 post-infection. (A) Hoechst nuclear stain (blue). Expression of (B) cleaved GSDME (red), (C) E-cadherin (epithelial cells; yellow), and (D) CD45 (immune cells; cyan). (E) Merged images. (A-E) Analysed with Image J software. Bronchiole (BR), alveolus (AL), and blood vessels (BV). Representative images at 40x magnification. Scale bar 100 µm. Percentage (%) of (F) cleaved GSDME$^+$, (G) E-cadherin (E-cad)$^+$ and cleaved GSDME$^+$, and (H) CD45$^+$ and cleaved GSDME$^+$ cells per field of view (FOV). (F-H) Five random fields of view were analyzed per animal using HALO software. Data are presented as mean % ± SD, with each data point representing an individual animal. P**<0.01, one-way ANOVA.

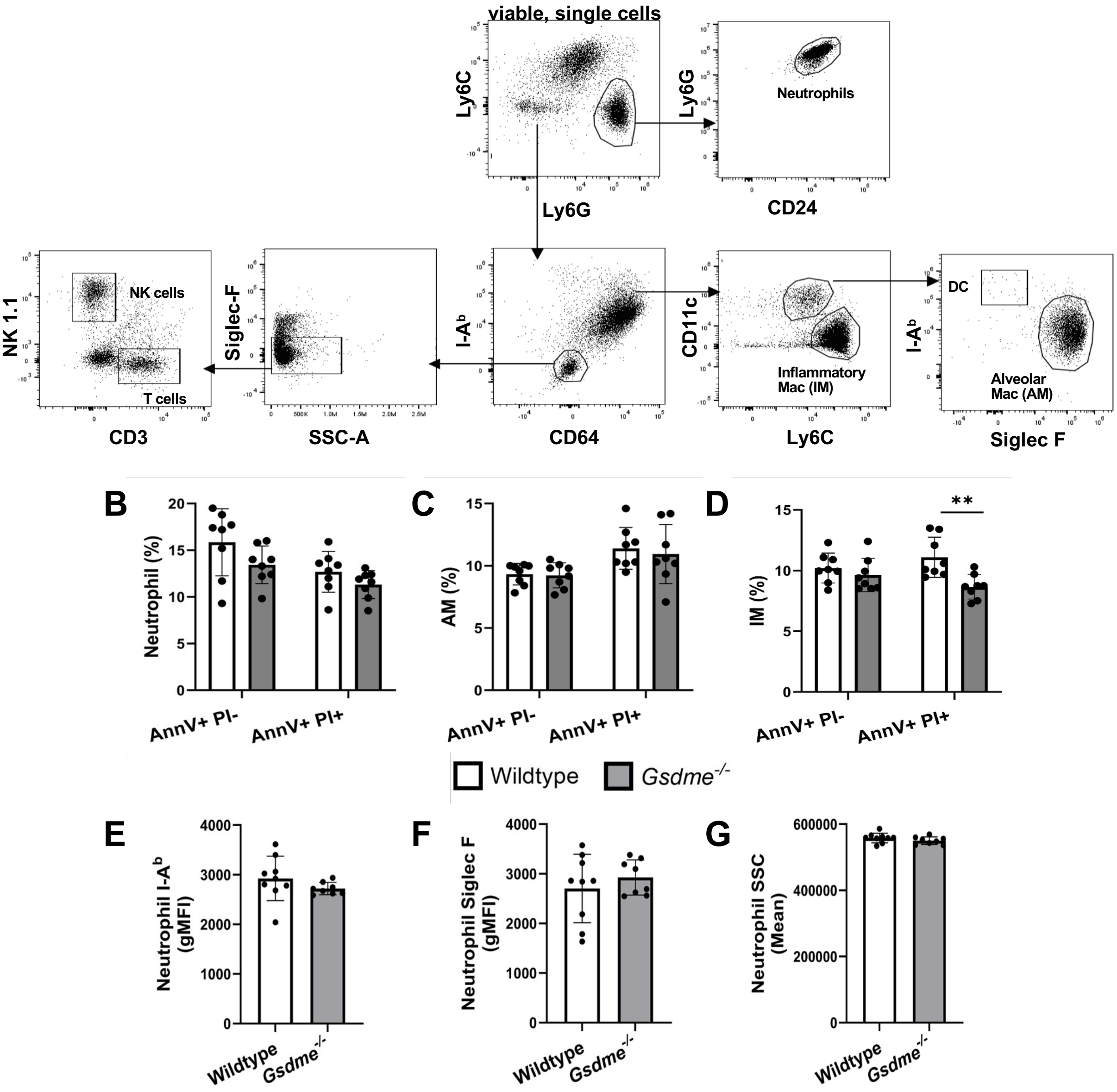

Figure S3. Infiltration of immune cells in the airways following IAV infection. (A-G) *Gsdme*$^{-/-}$ mice and wildtype littermates were infected with 10^4 PFU HKx31 IAV, and BAL was performed on day 3 post-infection, and BAL cells were analysed by flow cytometry. (A) Representative flow cytometry gating strategy for BAL cells. Percentage of (B) neutrophils, (C) alveolar macrophages (AM), and (D) inflammatory monocytes/macrophages (IM) that were Annexin V+ PI- (AnnV+ PI-) and Annexin V+ PI+ (AnnV+ PI+). Expression of (E) MHC class II (I-A^b) and (F) Siglec-F on neutrophils. Data are presented as geometric mean fluorescence intensity (gMFI). (G) Mean neutrophil side-scatter (SSC). (B-G) Data are presented as mean ± SD, with each data point representing an individual animal. ***P*<0.01, two-tailed, unpaired Student's *t* test. n=6-8 per group.

Full blots for Figure 1A

α-GSDME

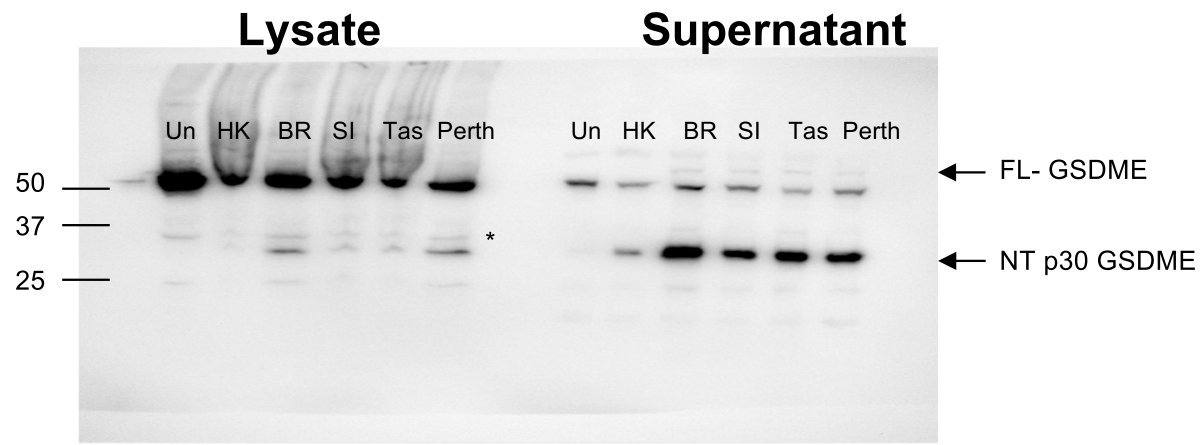

α-caspase 3

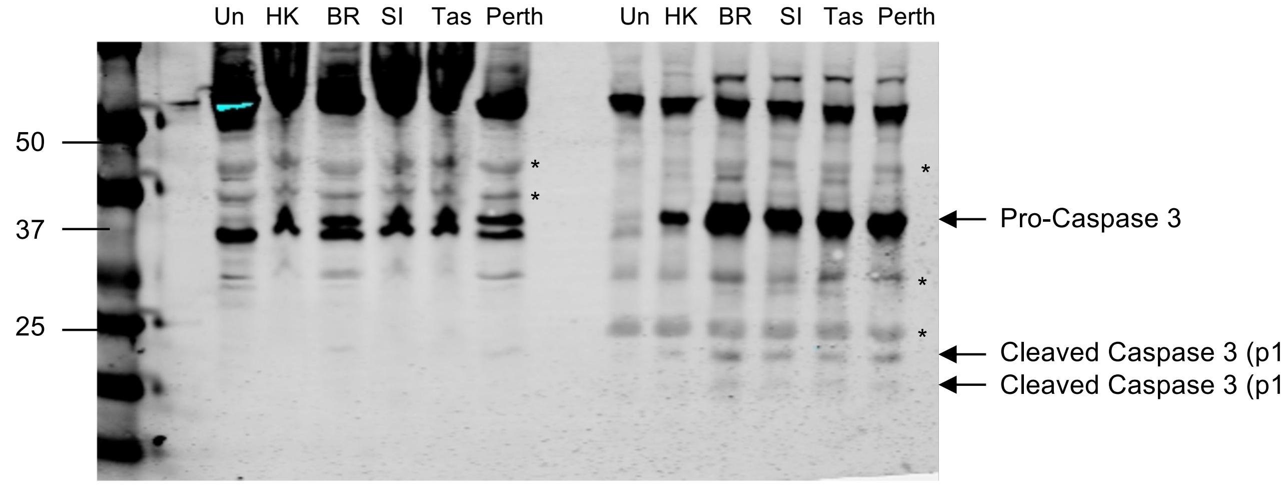

α-Tubulin

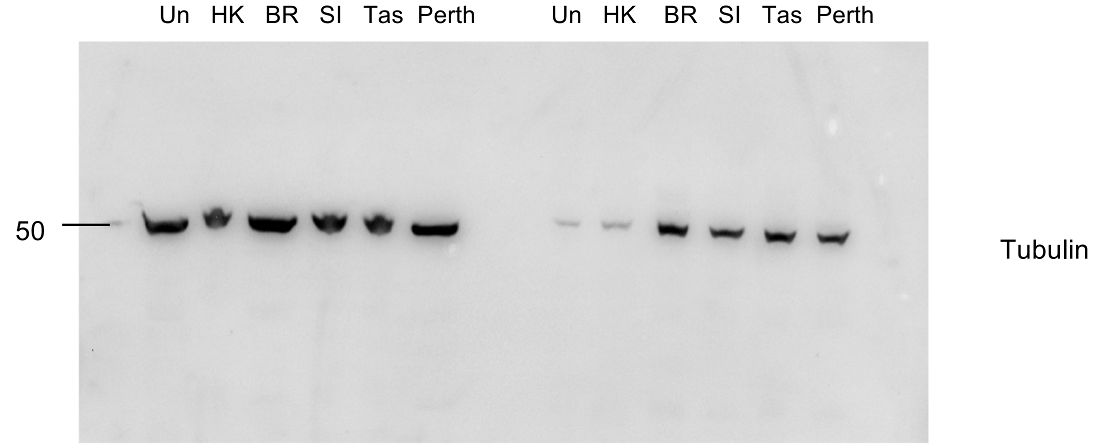

Full blots for Figure 2A - Lysate

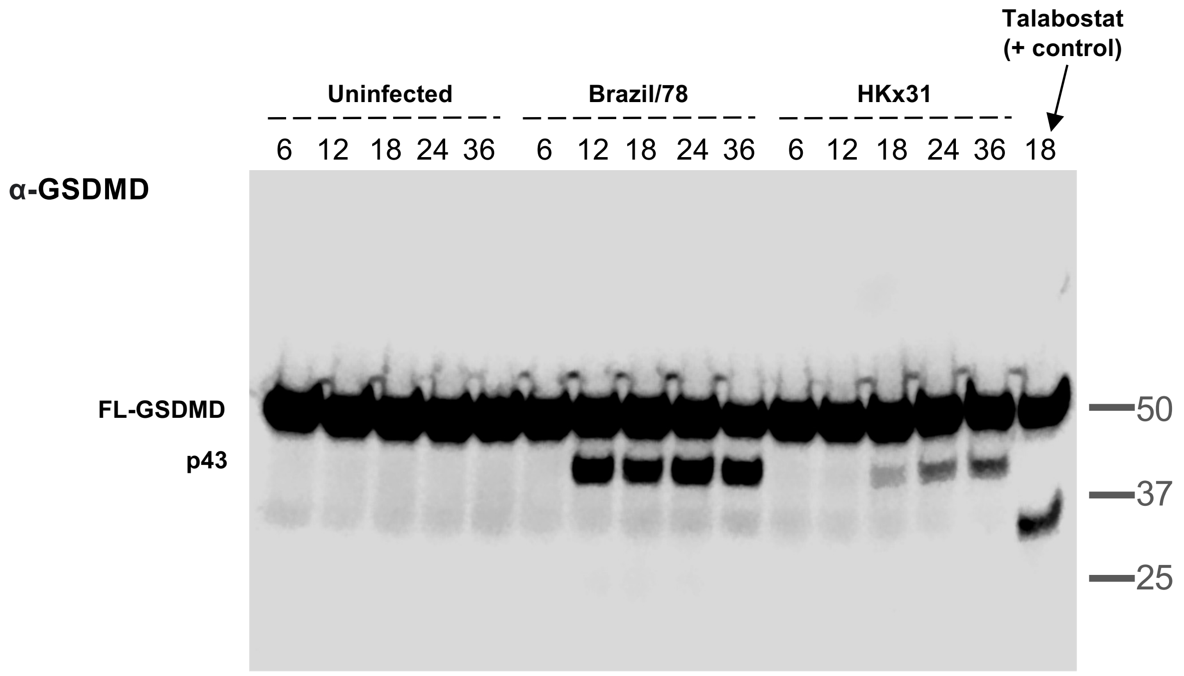

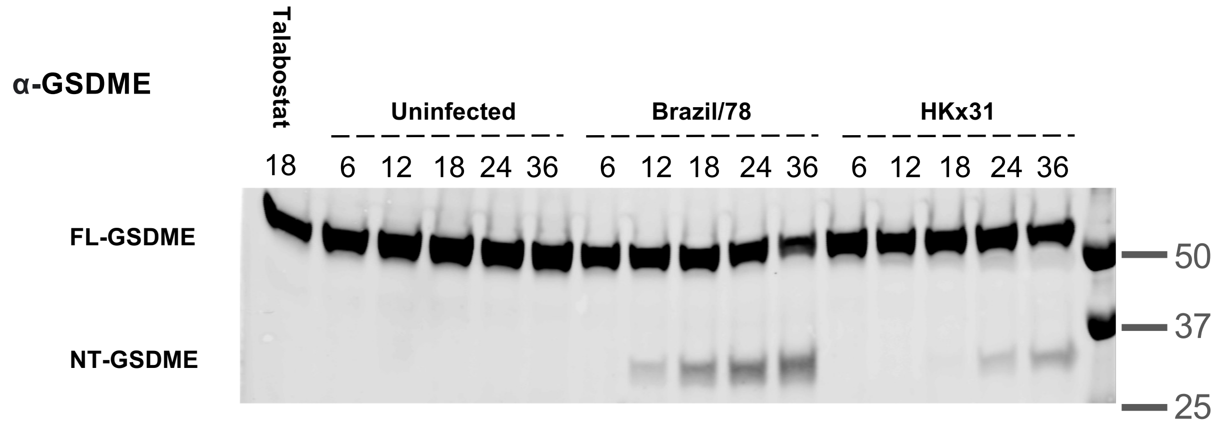

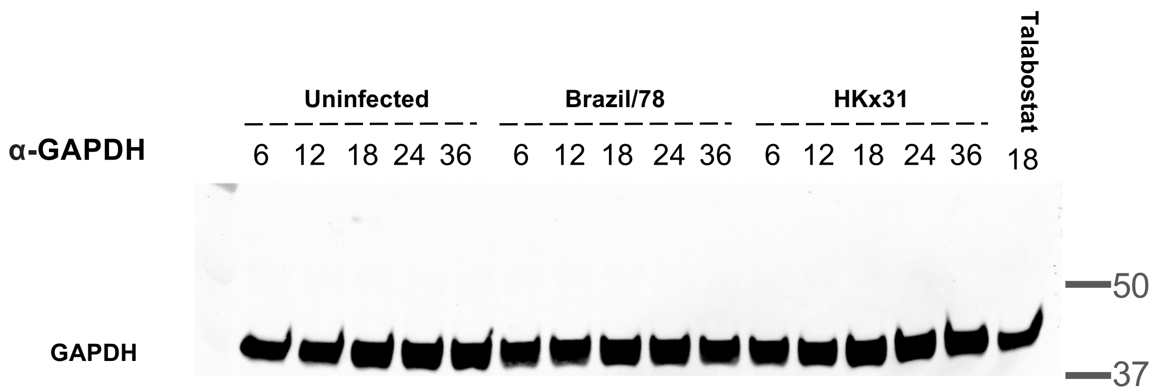

Full blots for Figure 2A - Supernatant

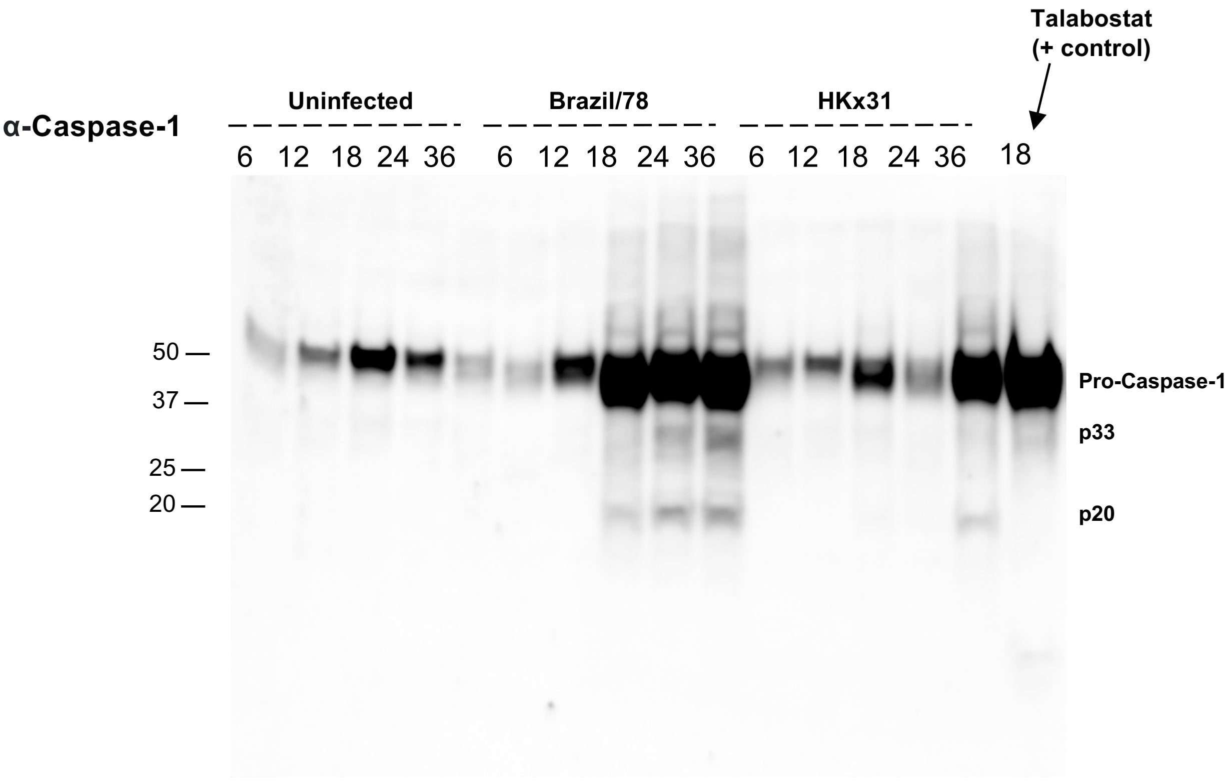

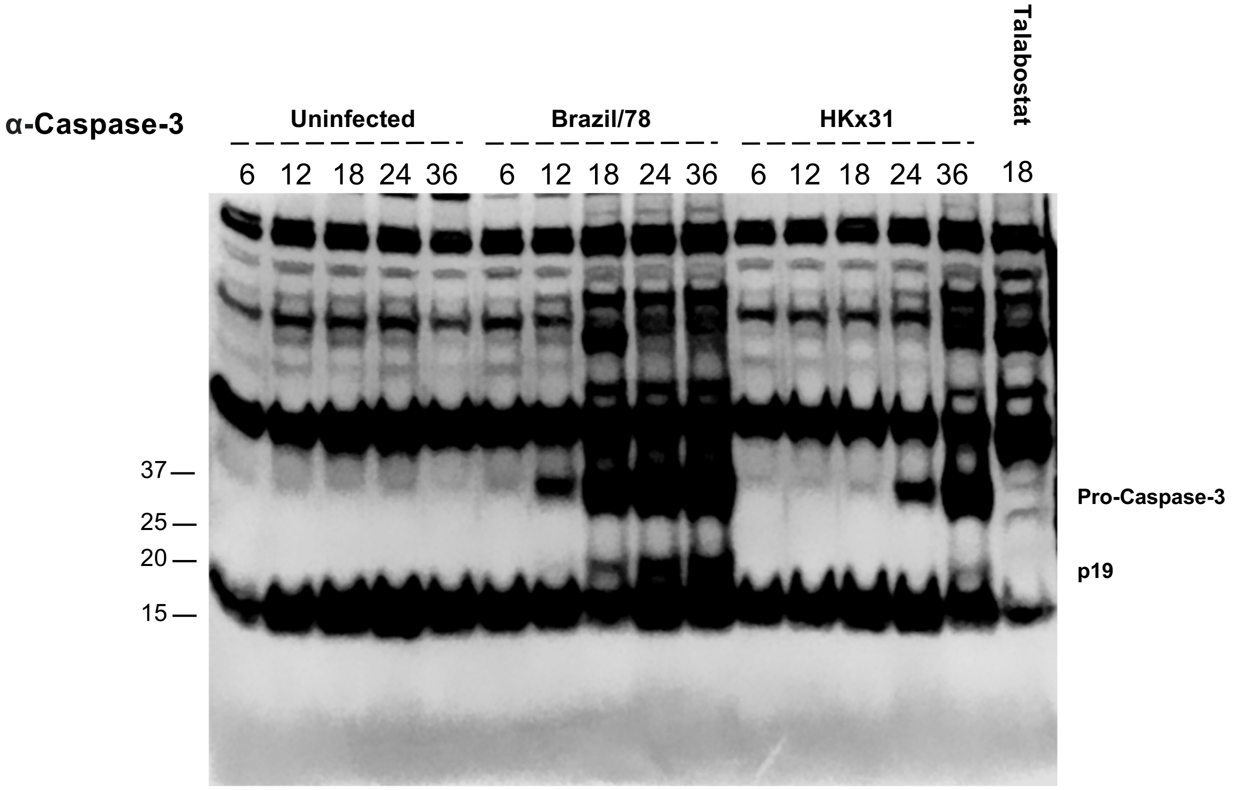

Full blots for Figure 2A - Supernatant

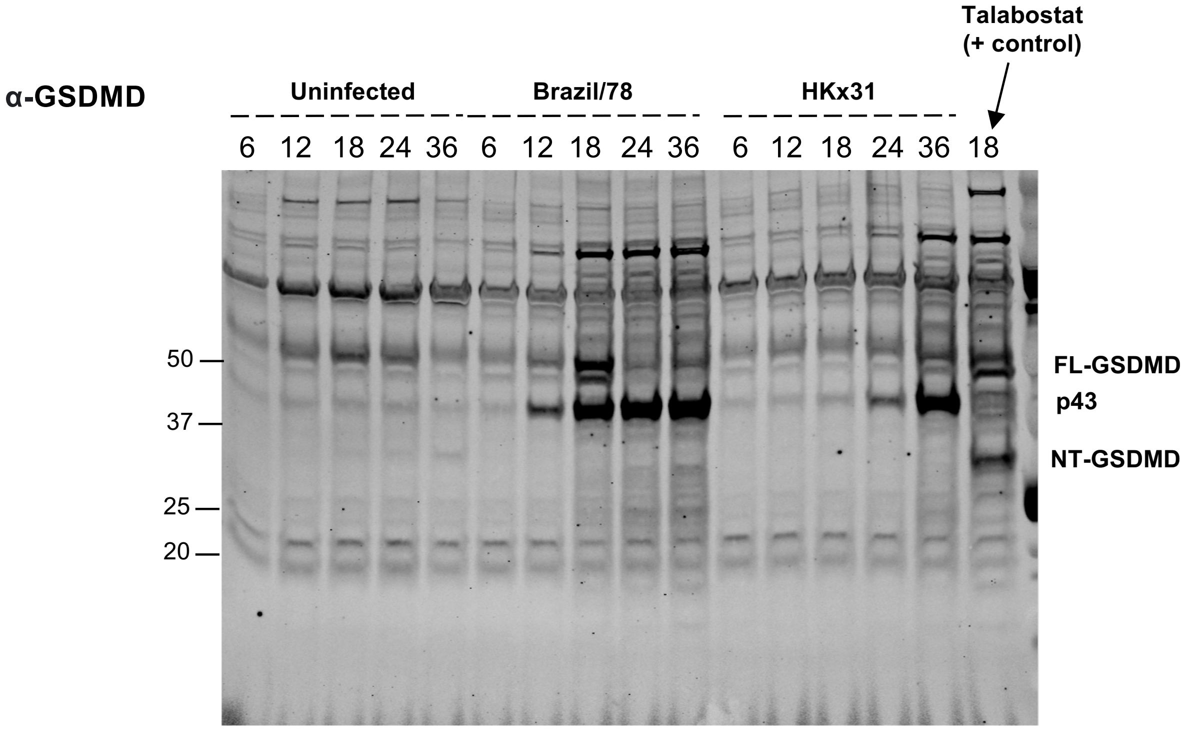

Full blots for Figure S1

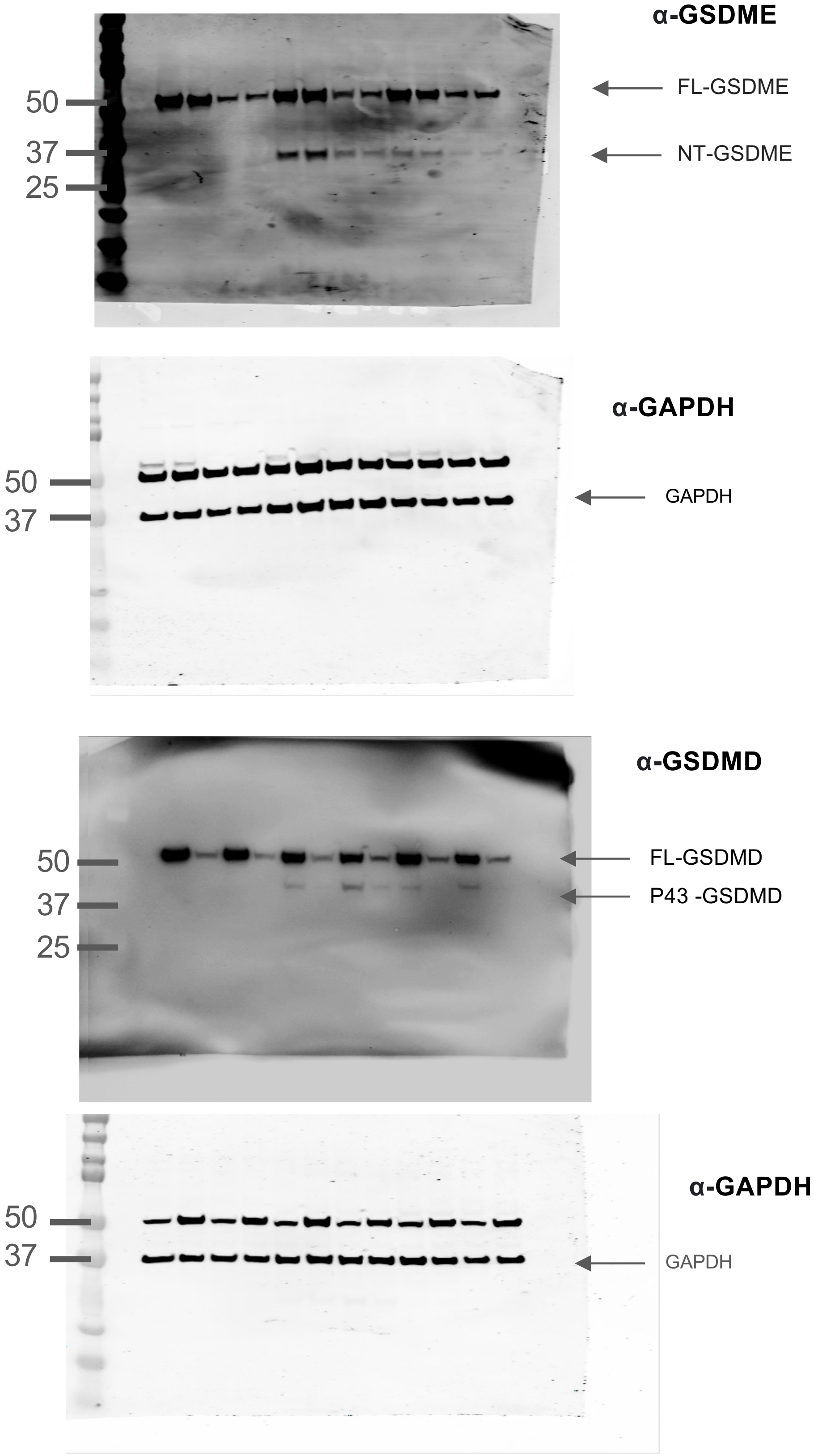

Supplement: Supplementary file 1 — Supplemental Material [file 41419_2025_7748_MOESM1_ESM.pdf]
